# The pathological Huntingtin CAG triplet expansion differentially affects the diagnosis of systemic and organ-specific autoimmune diseases

**DOI:** 10.3389/fimmu.2026.1689962

**Published:** 2026-05-08

**Authors:** Moritz Heyd, G. Bernhard Landwehrmeyer, Jan Lewerenz

**Affiliations:** Department of Neurology, Ulm University Hospital, Ulm, Germany

**Keywords:** autoimmune disease, Hashimoto thyroiditis, Huntington’s disease, psoriasis, rheumatoid arthritis

## Abstract

**Background:**

In Huntington’s disease (HD), signs of inflammatory activation are found in the brain, cerebrospinal fluid, and blood. HD monocytes are reported to be hyperreactive *in vitro.* Thus, HD mutation might affect the immune system.

**Aim:**

To explore the frequency of autoimmune diseases (AIDs) in HD mutation carriers (people with the HD mutation, PwHD) compared to control participants (CPs) as markers of immune dysfunction related to CAG triplet expansion in the Huntingtin (*HTT*) gene.

**Methods:**

Analysis of the Enroll-HD periodic dataset #5 (European sites) was conducted. Definite AIDs, coded using the abbreviated ICD-10 in the dataset for comorbidities, were identified. AIDs were grouped by organ specificity into arthropathy-dominant AIDs of musculoskeletal and connective tissues (arthropathic), as well as endocrine, dermatological, and gastrointestinal AIDs.

**Results:**

Although AID frequency was not different in PwHD (709/10,594; 6.7%) compared to CPs (176/2,477; 7.1%, *p* = 0.451), the AID subgroup distribution differed (*p* = 0.033) with endocrine AIDs being less frequent in PwHD [odds ratio (OR): 0.80; 95% confidence interval (95% CI) 0.68–0.95], while dermatological AIDs tended to be more common [OR (95% CI): 1.13 (0.94–1.38)]. These observations were explained by a reduced frequency of Hashimoto thyroiditis in PwHD [OR (95% CI): 0.69 (0.56–0.86)], while carriership of the HD mutation was associated with an increased risk of psoriasis [OR (95% CI): 1.27 (1.03–1.60)]. Among PwHD, those with an AID had lower CAG repeats [median (interquartile range): 42 (41–44)] than those without [43 (41–45), *p* < 0.0001]. When adjusted for sex and age, each extra pathological CAG repeat reduced the AID risk [OR (95% CI): 0.69 (0.61–0.78), *p* < 0.001]. The CAG dependency of the AID frequency among PwHD was mostly explained by arthropathic AID. In a well-defined early-manifest PwHD core group, each additional CAG repeat reduced the likelihood of an AID_arthro_ when adjusted for functional impairment, sex, and age at enrollment with an odds ratio of 0.57 (95% CI: 0.44–0.74, *p* < 0.0001).

**Conclusion:**

Both the presence and the exact size of the pathological CAG triplet expansion in the *HTT* gene differentially affect the frequency of certain AIDs. Our results support the idea that HD mutations affect immune function, but in a complex, disease-specific pattern.

## 1 Introduction

Huntington’s disease (HD) is an autosomal dominant neurodegenerative disease. The underlying mutation is a CAG triplet expansion in exon 1 of the Huntingtin (*HTT*) gene. While penetrance is incomplete at 36 to 39 CAG repeats, mutations above 39 CAG repeats are fully penetrant, with higher CAG repeats being associated with earlier onset and disease-related death (1). Although the mutant HTT protein is expressed in virtually all tissues, mutation-related cell loss is primarily observed in neurons of the central nervous system. Somatic expansion of the CAG repeats in the mutant *HTT* allele during a lifetime is a prerequisite for neurodegeneration (1). Mechanisms that ultimately induce neurodegeneration may include toxicity of mutant HTT mRNA, toxicity of the mutant HTT protein, including its aggregation, and repeat-associated non-AUG translation of the repeats (1).

In addition to the intraneuronal accumulation of HTT protein aggregates and neuronal loss, especially of the medium spiny neurons in the striatum (2), microglial activation is a neuropathological hallmark of HD (3). Multiple inflammatory markers including those of increased T-cell activation have been found elevated in HD cerebrospinal fluid (CSF) (4–7). However, HD-associated inflammation does not seem to be restricted to the brain. Both in motor-manifest people with the HD mutation (PwHD) and in mouse models of the disease, blood markers hint at a chronic systemic inflammatory state (8–10). In addition, it has been demonstrated that different aspects of the clinical phenotype in HD correlate differentially with blood cytokine levels (11). Although the exact role of inflammation in HD still remains to be defined (12), systemic inhibition of TNF-α signaling decreased systemic inflammation and ameliorated brain volume loss in an HD mouse model (13). Blood-derived monocytes of HD patients have been demonstrated to be more responsive to pro-inflammatory stimuli regarding cytokine release *in vitro* (9, 14). Animal studies suggest that this is a direct result of mutant *HTT* gene expression in these cells (15).

Inflammatory activation of cells of the innate immune system is one of the prerequisites of activating the adaptive immune system (16). Autoimmune diseases (AIDs) represent mostly chronic diseases that result from a breach of the adaptive immune system’s self-tolerance against specific autoantigens (17). Autoimmunity results in organ dysfunction and/or tissue destruction due to autoreactive T cells or T and B cells. Common AIDs include autoimmune thyroiditis, especially Hashimoto thyroiditis (HT), diabetes mellitus type 1, rheumatoid arthritis (RA), and psoriasis (18). Many AIDs show distinctive age and sex association (19). Genetic risk factors for several AIDs are certain HLA alleles with the unique capacity to present specific peptides of autoantigens to cells of the adaptive immune system (17).

As HD mutation has been described to render cells of the innate immune system hyperreactive (9, 14), we asked whether the reported hyperreactivity in PwHD is associated with an increased risk for autoimmunity. To this end, we identified AIDs among the comorbidities in PwHD and control participants (CPs) in the Enroll-HD dataset. Enroll-HD is a worldwide registry for presymptomatic as well as motor-manifest PwHD as well as family CPs who all undergo detailed clinical and *HTT* genetic characterization, including documentation of comorbidities not associated with HD (20).

## 2 Materials and methods

### 2.1 The Enroll-HD periodic dataset

The Enroll-HD (ClinicalTrials.gov ID: NCT01574053) periodic dataset (PDS) 5 was downloaded from the Enroll-HD website (https://www.enroll-hd.org/for-researchers/datasets/) following the submission of an abstract for the project. The full description of the PDS can be found at https://www.enroll-hd.org/for-researchers/pds-data-explorer/. Enroll-HD (NCT01574053) is a global prospective registry for PwHD as well as CPs, usually family members (~20%) (20). In the PDS, study participants are de-identified. Although data entry and coding in Enroll-HD are performed uniformly at all sites, monitoring and quality control are not. In Europe, monitoring is performed by the language-area coordinators who are a bridge between the European Huntington’s Disease Network and the Enroll-HD sites. In contrast to the site in North America, monitoring in Europe is performed with access to the participants’ medical files if they are patients of the respective HD center.

### 2.2 Identification of ICD-10 codes representing autoimmune diseases

Upon enrolment in Enroll-HD, comorbidities are entered as text into the “Comorbid” form. The text is converted by the Enroll-HD central coding team into ICD-10 codes (https://www.who.int/standards/classifications/classification-of-diseases) with the limitation that ICD-10 codes in the PDS are restricted to one digit following the full stop. This leads to some ambiguity in the text-to-code conversion. In some cases, the second digit after the full stop in the ICD-10 code unequivocally may indicate an AID, while alternative second digits after the full stop may indicate other pathophysiologies. The ICD-10 was thus thoroughly reviewed for codes that, with one digit following the full stop, only clearly stand for an AID. However, individual codes do not have a 100% specificity for an autoimmune disease; for example, “arthritis, unspecified” (M13.9), aka undifferentiated arthritis, does not per se exclude arthritis of metabolic (gout) or infectious origin. However, in most cases, the diagnosis is used to code for autoimmune arthritis until a more specific diagnosis is established as supported by the fact that disease-modifying antirheumatic drugs are prescribed in >85% of patients with this diagnosis (21). Clearly ambiguous abbreviated codes were not counted as representing an AID (**Supplementary Table 1**). Although the “Comorbid” form is supposed to be updated at each annual Enroll-HD visit, we assumed that data entry was most complete at enrolment and including AID diagnoses coded at later visits will introduce a bias regarding the number of visits of an individual. Thus, comorbidities documented upon enrolment were analyzed.

### 2.3 Grouping of AIDs and Enroll-HD participants

AIDs can affect virtually all organs. In general, AIDs can be categorized as systemic and organ-specific. In organ-specific AIDs, the autoantigens tend to be restricted to one specific organ or cell type, while in systemic AIDs, the autoantigen is usually much more abundant. However, many “systemic” autoimmune diseases tend to be predominated by vascular, connective tissue, and musculoskeletal pathologies. Thus, we grouped the AIDs coded in the ICD according to the predominant organ specificity (**Supplementary Figures 1**–**9**). Of note, we decided to introduce some exceptions: as melanocytes are neuronal crest-derived cells and share many similarities with endocrine cell types (22), we grouped vitiligo (L80) with the other endocrine AIDs. In addition, although arthropathic psoriasis (L40.5) affects the joints, it remained grouped with psoriasis as a dermatological AID. We acknowledge that this might create some ambiguity as undifferentiated arthritis (M13.9) might later be classified as psoriatic arthritis (L50.5/M07.3/M09.0). Chapter XIII comprises AIDs of musculoskeletal and connective tissues, including vasculitides, systemic sclerosis, lupus erythematosus, Sjögren’s syndrome and myositides, RA, ankylosing spondylitis, and other forms of autoimmune arthritis. As this group was perceived as quite heterogeneous, it was divided into two groups: AIDs of musculoskeletal and connective tissues, where connective/soft-tissue involvement dominates the clinical presentation (**Supplementary Figure 7**), and those that usually present as arthropathy-dominant AIDs comprising RA, ankylosing spondylitis, and other forms of autoimmune arthritis, with the exception of arthropathic psoriasis (**Supplementary Figure 8**).

For the analyses of the whole cohort, if two or more AIDs of different AID categories were documented, only the one of the larger groups was counted. For the analyses within one AID category, all participants with a diagnosis assigned to this category were used. However, if those participants were diagnosed with two different AIDs of the same category, the more frequent AID was counted. Exceptions were the arthropathy-dominant AIDs of musculoskeletal and connective tissues (arthropathic AID), where when one specific, e.g., RA, and a more unspecific code, e.g., unspecified arthritis (for more information, see **Supplementary Figure 8**) were documented simultaneously, the more specific code was chosen. For the analysis of HT, RA, or psoriasis frequencies, the exact number of documented diagnoses irrespective of other AIDs was used.

### 2.4 Ethical considerations

The Enroll-HD study is funded and run by CHDI and is conducted under the oversight and approval of multiple Institutional Review Boards (IRBS), with each site having its own consent form and process overseen by the local IRB. As consented by the participants, this research is performed based on de-identified data made available as PDSs by CHDI. Our local IRB thus confirmed that the current research does not need an additional local IRB review.

### 2.5 Statistical analysis

Data were analyzed using the R programming language (R Core Team (2022). R: A language and environment for statistical computing. R Foundation for Statistical Computing, Vienna, Austria. URL: https://www.R-project.org) and GraphPad Prism, version 10.4.1 (GraphPad Prism Software, Boston, MA, USA). For bivariate statistics with continuous variables, Mann–Whitney *U* tests were used; for categorical variables, Fisher’s exact and chi-square tests were used as indicated. In case of multiple pairwise Fisher’s exact tests performed on one dataset, the *p*-values were Bonferroni-adjusted. For multiple logistic regression to analyze binary variables in the whole cohort, we used odds ratios to interpret the direction of correlations. The impact of independent variables was described as estimated parameters with standard deviation and *p*-value of the *z*-test to analyze their significance. As a general measure of model fit, Tjur-adjusted *R*² was used. The significance level was set to *α* = 0.05 for all analyses. As independent variables, we included in our regression analysis sex and age because of the differences in sex and age distribution of different AIDs (17). Dichotomous variables were effect-coded as 1 and −1, and continuous variables were *z*-standardized. To identify significant interactions between independent variables, we added interactions step by step to the original model, checking for improvements of predictive power, using the difference of the residual deviance of the models in a chi-square test and *p*-value as well as Akaike and Bayesian information criterion (AIC/BIC) to compare the models. For multiple logistic regression analysis (MLRA) using a very narrowly defined core cohort to analyze the CAG dependency of arthropathic AID, no *z*-standardization was feasible, and thus, no analysis of interaction was performed.

Due to the retrospective nature of the study, all statistical analyses were exploratory.

## 3 Results

### 3.1 Categorization of autoimmune comorbidities documented in the Enroll-HD periodic dataset

Review of the comorbidities coded using an abbreviated ICD code with one digit following the full stop only as in the Enroll-HD PDS identified 210 different codes representing 83 distinct AIDs. Only six ICD-10 codes encoding an AID turned ambiguous after omission of the second digit following the full stop (D68.31, Acquired Hemophilia A; D68.38, Hemorrhagic disorder due to other antibodies; D69.53, Heparin-induced thrombocytopenia Type II; K83.00, Primary sclerosing cholangitis; K86.10, Autoimmune pancreatitis, **Supplementary Table 1**). The codes for definite AIDs were classified into nine groups depending on the tissues or cell lineage affected: 1. AIDs of the blood and immune system (chapter III, D50–D90, **Supplementary Figure 1**); 2. endocrine AIDs (chapter IV, E00–E90, **Supplementary Figure 2**); 3. the nervous system (chapter VI, G00–99, **Supplementary Figure 3**); 4. rheumatoid fever (chapter IX, I00–I99, **Supplementary Figure 4**); 5. AIDs of gastrointestinal tract (chapter XI, K00–K93, **Supplementary Figure 5**); 6. dermatological AIDs (chapter XII, L00–L99, **Supplementary Figure 6**); 7./8. AIDs of the musculoskeletal and connective tissues (chapter XIII, M00–99) divided into two groups: 7. arthropathic AIDs including RA (**Supplementary Figure 7**) and 8. non-arthropathy-dominant AIDs of the musculoskeletal and connective tissues (**Supplementary Figure 8**); and finally, 9. AIDs of the kidney (chapter XIV, N00–99, **Supplementary Figure 9**).

Among the 13,071 European participants included in the Enroll-HD PDS, 10,594 (81%) were PwHD, while 2,477 were CPs (19%, **Table 1**). Within the PDS, 95 of the 210 AID codes (45%) were used for coding AID comorbidities upon enrolment (**Supplementary Appendix Table À**). In total, 965 codes indicating AIDs were identified in 885 of all 13,071 participants (6.8%, **Supplementary Appendix Table A**). More than one AID was documented in 77 (8.7%); in 20 participants, two codes indicating the same AID was recorded (2%, **Supplementary Appendix Tables B, C**). Of the ambiguous ICD-10 codes, only K83.0 and K86.10 were used one and two times, respectively (*N* = 3 in total, **Supplementary Table 2**).

**Table 1:** Description of the study cohort.

Characteristic	CP (*N* = 2,477)	PwHD (*N* = 10,594)	*p*-values
Sex, f:m, *N* (%)	1,494:983 (60:40)	5,629:4,965 (53:47)	<0.01^a^
Age (years), median (IQR)	45 (33–56)	48 (38–58)	<0.01^b^
CAG, median (IQR)		43 (41–45)	
TFC, median (IQR)		11 (8–13)	
Stadium, premanifest/manifest (stages I:II : III:IV:V), *N* (%)		2,802 (26)/3,842:2,356:1,164:286:131 (36:22:11:3:1)	

CP, control participants; PwHD, people with the HD mutation; f, female; m, male; IQR, interquartile range; CAG, CAG triplet repeat expansion, expanded allele; TFC, total functional capacity; stages I–V, functional disease stages according to Shoulson and Fahn.

^a^ Fisher’s exact test.

^b^ Mann–Whitney *U* test.

### 3.2 Endocrine autoimmunities are documented less often, while dermatological autoimmune diseases tend to occur more often in people with the Huntington mutation when compared to control participants

**Figure 1:**
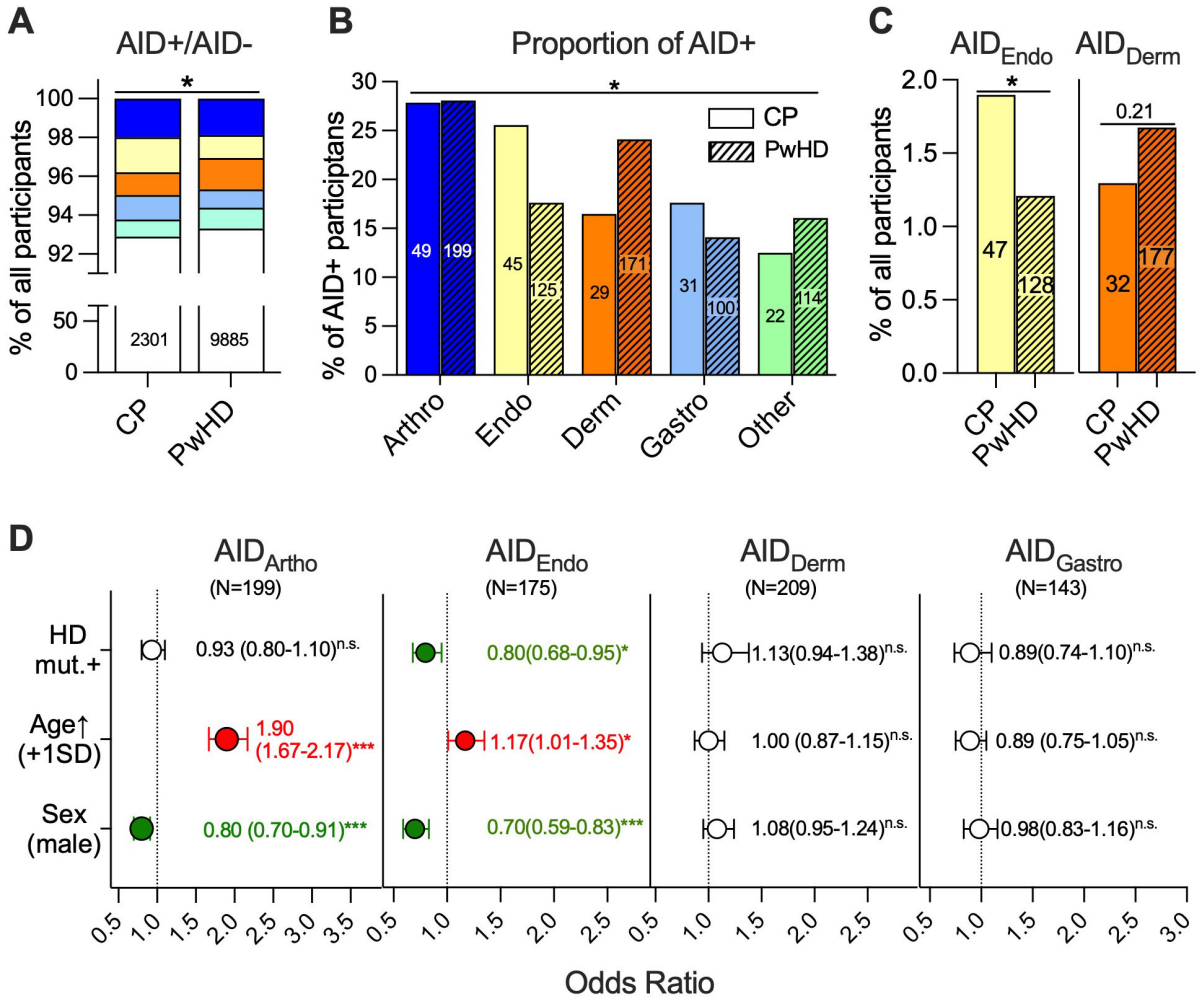
Endocrine autoimmune diseases are less frequent in HD mutation carriers compared to control participants, while there is a tendency to an increased frequency of dermatologic autoimmune disease. **(A)** Frequency distribution of European Enroll-HD control participants (CPs) and HD mutation carriers (people with the HD mutation, PwHD) without an autoimmune disease (AID) documented as comorbidity upon enrolment (clear) or with an AID of one of the five categories [blue: arthropathy-dominant AIDs of musculoskeletal and connective tissues (Arthro), light yellow: endocrine AIDs (Endo), light blue: gastrointestinal AIDs (Gastro), orange: dermatological AID (Derm), light green: other AIDs (Other)] documented. **(B)** Proportion of participants with an AID of the five categories among CPs (no pattern) and PwHD (oblique stripes) with an AID documented. The number of participants is given within the bars. **(C)** Frequency of endocrine AIDs (AID_Endo_) and dermatological AIDs (AID_Derm_) among all CPs and PwHD. The number of participants is given within the bars. **(D)** Forest plots of the odds ratios for documentation of an arthropathy-dominant AID of musculoskeletal and connective tissues (AID_Arthro_), AID_Endo_, AID_Derm_, and gastrointestinal AID (AID_Gastro_) for carriership of the HT mutation (HD mut.+) with higher age in years [age↑ (years)] per standard deviation (SD, 13.9 years) and male sex as influencing variables estimated by multiple logistic regression analysis. The symbols and error bars depict the means and 95% confidence intervals, which are also given right next to the symbols. Increased risk: red, decreased risk: green. Statistical analysis was performed using the chi-square test **(A)**, Fisher’s exact test **(B, C)**, and multiple logistic regression **(D)** with the identification of significant interactions between independent variables and dichotomous variables being effect-coded as −1 and 1 and continuous variables being *z*-standardized; n.s. = not significant, *p < 0.05, **p < 0.01, ***p < 0.001.

Among the 2,477 family CPs, 176 (7.1%) were documented as AID-positive, while among the 10,594 HD mutations carriers, 709 (6.7%) were documented as AID-positive (**Supplementary Appendix Table D**). Of those, seven of the 176 CPs (4.0%) and 23 PwHD participants (3.2%) were documented with a second AID of a less frequent category other than that to which they were assigned (**Supplementary Figure 10A**). An additional AID of a less frequent category was not more frequent in PwHD when compared to CPs (**Supplementary Figures 10A, B**). There was no difference in the frequency of participants with at least one AID diagnosis between PwHD and CPs (Fisher’s exact test, *p* = 0.451). After prioritizing the most common AID groups among the participants with AIDs of more than one AID category, AID-positive participants were assigned the most frequent AID category—arthropathic AIDs (*N* = 248, 28%)—followed by the dermatological (*N* = 200, 23%), endocrine (*N* = 168, 19%), and gastrointestinal (*N* = 133, 15%) AID categories (**Supplementary Appendix Table D**). All the other categories were coded less often than 50 times and combined to the category “other AID” [“other AID” total: *N* = 136 (15%); non-arthropathy-dominant AIDs of the musculoskeletal and connective tissues: *N* = 33 (4%), neurological AIDs: *N* = 39 (4%), rheumatoid fever: *N* = 26 (3%), AIDs of the blood and immune system: *N* = 25 (3%), AIDs of the kidney: 5 (1%)]. The distribution of participants assigned to these categories including AID-negatives differed among the PwHD and CPs (chi-square test, *p* = 0.044, **Figure 1A**), as did their distribution among the AID categories when only AID-positive participants were considered (Fisher’s exact test, *p* = 0.034, **Figure 1B**). Analyzing the distribution of all AID category frequencies without omitting the less frequent in case of AIDs of two categories in a participant did not change this result (Fisher’s exact test, *p* = 0.035, **Supplementary Figure 10C**). In fact, the percentage of all participants with endocrine AIDs documented either alone or with an AID of another category was 1.6-fold lower in PwHD (128/10,594, 1.2%) compared to CPs (47/2,477, 1.9%, *p* = 0.014; **Figure 1C**, left panel). In contrast, the percentage of participants with a dermatological AID documented was 1.3-fold higher in PwHD (177/10,594, 1.7%) compared to CPs (32/2,477, 1.3%), although this difference failed to reach statistical significance (*p* = 0.21; **Figure 1C**, right panel). As the prevalence of different AIDs is age- and sex-dependent (17) and both sex distribution and age at enrolment were different in PwHD compared to CPs (**Table 1**), we next analyzed how the carriership of the HD mutation influenced the likelihood of an AID of the four major categories—arthropathic, endocrine, dermatological, and gastrointestinal AIDs—using MLRA, taking these differences into account (**Figure 1D**). For each MLRA, irrespective of comorbidities with AIDs of more frequent categories, all participants with at least one AID of the respective category were counted. Therefore, the group sizes for each analysis became slightly higher (endocrine AIDs: *N* = 175 vs. *N* = 170, dermatological AIDs: *N* = 209 vs. *N* = 200, gastroenterological AIDs: *N* = 143 vs. *N* = 131). While carriership of the HD mutation did not affect the likelihood of an arthropathic AID [odds ratio (OR), 95% confidence interval (95% CI): 0.93 (0.80–1.10); *p* = 0.401] or gastroenterological AID [OR (95% CI): 0.89 (0.74–1.10); *p* = 0.261], it was associated with a decreased likelihood that a participant was documented with an endocrine AID [OR (95% CI): 0.80 (0.68–0.95); *p* = 0.011]. There was a tendency that carriership of the HD mutation was associated with an increased likelihood of a participant being documented with a dermatological AID [OR (95% CI): 1.13 (0.94–1.38); *p* = 0.120]. Age and sex were confounding factors in arthropathic and endocrine AIDs only (**Figure 1D**).

### 3.3 When compared to control participants, Hashimoto thyroiditis is documented less frequently and psoriasis more frequently in people with the Huntington mutation

**Figure 2:**
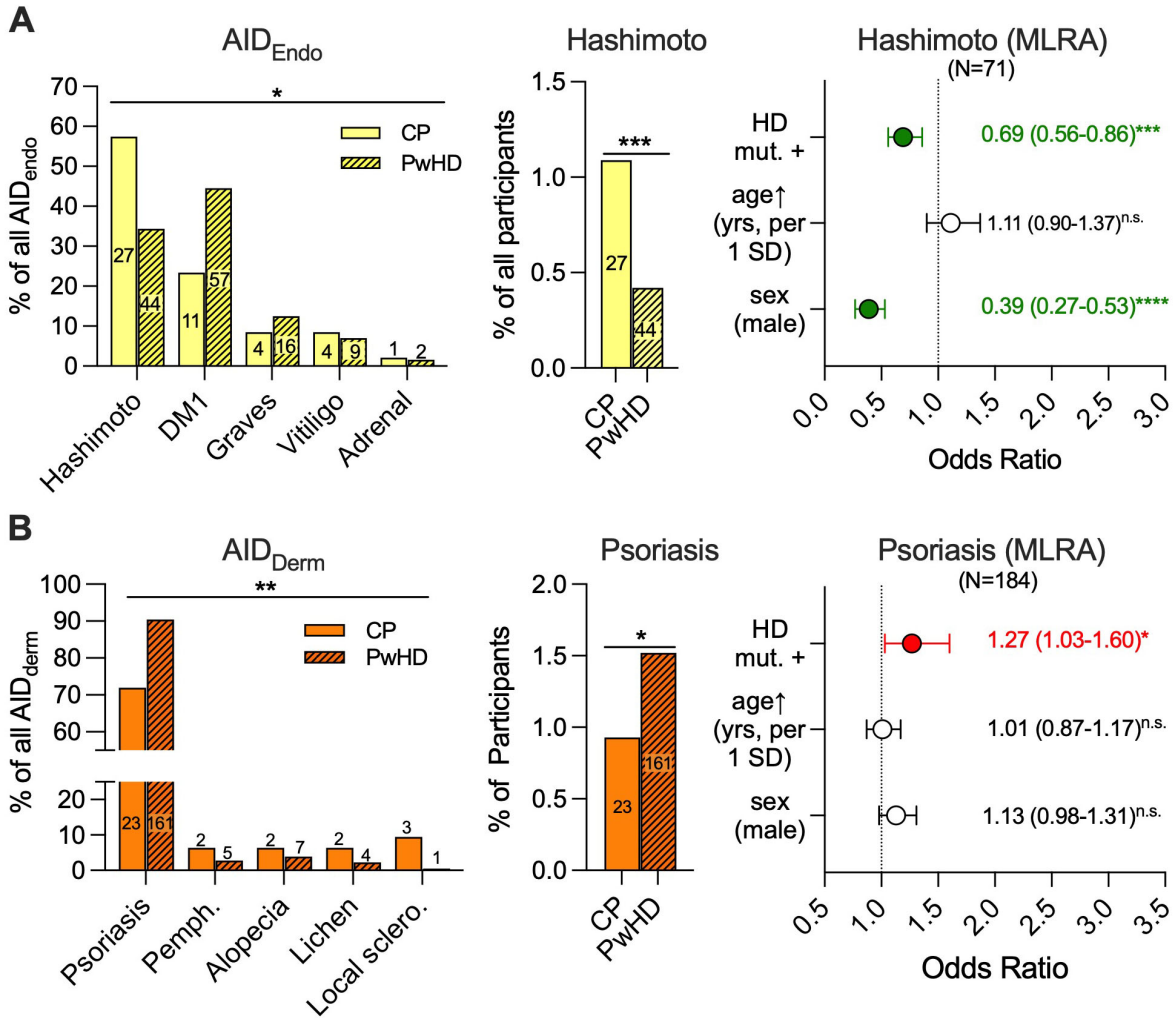
Hashimoto thyroiditis is less frequently documented as a secondary diagnosis in Enroll-HD participants carrying the HD mutation upon study enrolment compared to control participants, while psoriasis is more frequent. (Left panels) Frequency distribution of different specific diagnoses among Enroll-HD control participants (CPs, clear) and HD mutation carriers (people with the HD mutation, PwHD, oblique stripes) with **(A)** endocrine autoimmune diseases (AID_Endo_; Hashimoto = Hashimoto thyroiditis, DM1 = diabetes mellitus type 1, Graves = Graves’ disease, Adrenal = autoimmune adrenal insufficiency) or **(B)** dermatological AIDs (AID_Derm_; Pemph. = blistering skin diseases, Local. sclero. = localized sclerodermia). (Middle panels) Absolute frequency of HT **(A)** and psoriasis **(B)** in CPs and PwHD. (Left and middle panels) The absolute numbers of participants are given within the bars. (Right panels) Odds ratios of the diagnosis of HT **(A)** or psoriasis **(B)** documented as comorbidities estimated by multiple logistic regression analysis (MLRA) with carriership of the HD mutation (HD mut. +), higher age in years [age↑ (years)] for each standard deviation (SD, 13.9 years), and male sex as influencing variables. The symbols and error bars depict the means and 95% confidence intervals, which are also given right next to the symbols. Increased risk: red, decreased risk: green. Statistical analysis was performed using Fisher’s exact tests (left and middle panels) and MLRA with the identification of significant interactions between independent variables and dichotomous variables being effect-coded as −1 and 1 and continuous variables being *z*-standardized; n.s. = not significant, *p < 0.05, **p < 0.01, ***p < 0.001, ****p < 0.0001.

We then went into more detail to identify which diagnoses underlie the effects seen for the participants with endocrine and dermatological AIDs, also including those with double AID diagnoses with an additional AID of a more abundant category (**Supplementary Appendix Table C**). When each participant with at least one endocrine AID documented was assigned to the group of the more frequent endocrine AID, those assigned to the Hashimoto thyroiditis (HT) group represented the largest subgroup among those with an endocrine AID among all the participants [71/13,071 (0.5%)], followed by diabetes mellitus type 1 [68/13,071 (0.5%)] and Graves’ disease [20/13,071 (0.2%)], while vitiligo and autoimmune adrenal insufficiency were documented only very infrequently [vitiligo: 13/13,071 (0.1%); adrenal insufficiency: 3/13,071 (0.02%)]. The relative proportions of these specific endocrine AIDs were significantly different when PwHD were compared to CPs with endocrine AIDs (**Figure 2A**, left panel; Fisher’s exact test, *p* = 0.041). This did not change when the frequencies of all endocrine AIDs irrespective of double diagnoses with a more abundant endocrine AIDs were analyzed (Fisher’s exact test, *p* = 0.034, **Supplementary Figure 10D**). The proportion of participants diagnosed with HT was lower among PwHD compared to CPs [44/10,594 (0.4%) vs. 27/2,477 (1.1%), Fisher’s exact test, *p* < 0.001; **Figure 2A**, middle panel]. As for HT, the descriptive diagnosis of hypothyroidism as a possible consequence of HT without HT itself being documented was also coded less frequently in PwHD when compared to CPs (**Supplementary Figure 11A**). Among the combined cohort of study participants with HT or hypothyroidism, no bias toward the less specific diagnosis of hypothyroidism was observed in CPs [CPs vs. PwHD: 72/99 (73%) vs. 203/247 (82%), Fisher’s exact test, *p* = 0.059; **Supplementary Figure 11B**]. Thus, we did not find any evidence of a relevant coding bias toward more unspecific diagnoses in CPs who are not patients of the respective study centers compared to PwHD who mostly are. Next, we employed MLRA to account for the effects of sex and age as confounders for HT. This analysis confirmed that the presence of the HD mutation significantly decreased the likelihood of a participant being diagnosed with HT at enrolment [OR (95% CI): 0.69 (0.56–0.86), *p* < 0.001; **Figure 2A**, right panel].

Among the total group with dermatological AIDs, psoriasis was by far the most frequent specific diagnosis [183/13,071 (1.4%)], followed by bullous dermatoses [7/13,071 (0.05%)], while other diagnoses were exceedingly rare [alopecia: 9/13,071 (0.06%), lichen: 6/13,071 (0.05%), morphea: 4/13,071 (0.03%)]. None of the participants with an AID of the dermatological AID category had two of the abovementioned diagnoses. The distribution of psoriasis, bullous dermatoses, other dermatological AIDs, and no dermatological AID was significantly different among PwHD when compared to CPs (Fisher’s exact test, *p* = 0.007; **Figure 2B**, left panel). Among the total cohort of 10,594 PwHD, participants diagnosed with psoriasis (*N* = 161, 1.5%) were 1.3-fold more frequent when compared to the 2,477 CPs, of which 23 (0.9%) had psoriasis documented as comorbidity (Fisher’s exact test, *p* = 0.023; **Figure 2B**, middle panel). MLRA confirmed that the presence of the HD mutation increased the likelihood for a participant to be documented with psoriasis as comorbidity [OR (95% CI): 1.27 (1.03–1.60), *p* = 0.033; **Figure 2B**, right panel]. In summary, these analyses suggested that the presence or absence of the HD mutation differentially affected the frequency to AIDs. Specifically, HT seems to occur less often and psoriasis more often in PwHD compared to CPs.

### 3.4 The higher CAG repeats in people with the Huntington mutation are associated with fewer arthropathic autoimmune diseases of the musculoskeletal system and connective tissues

**Figure 3:**
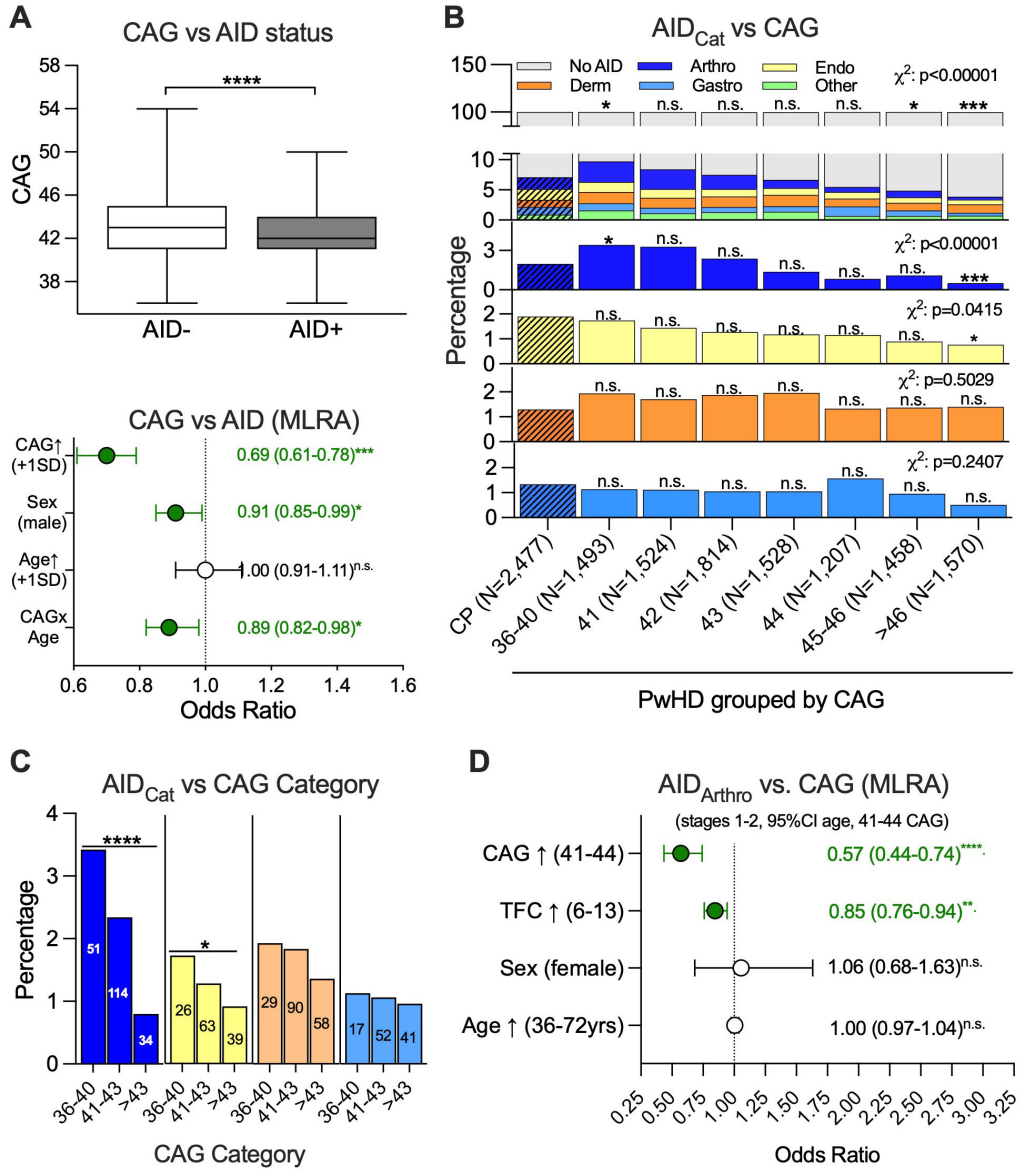
A CAG-dependent decrease in the frequency of arthopathy-dominant autoimmune disease of the musculoskeletal and connective tissues largely explains why larger pathological CAG triplet expansions are associated with a decreased frequency of autoimmune diseases in HD mutation carriers. [**(A)**, upper panel] Pathological CAG triplet length in HD mutation carriers (people with the HD mutation, PwHD) with autoimmune disease (AID+, gray fill) in comparison to those without an autoimmune disease (AID−, empty). The Tukey-style box-and-whisker plot indicates the median and interquartile range. [**(A)**, lower panel] Forest plot showing the odds ratios depicted as mean and 95% confidence interval estimated by multiple logistic regression analysis (MLRA) of an AID being diagnosed among PwHD with each standard deviation (SD) higher CAG triplet repeats (1 SD = 9.9 CAG, CAG↑), male sex, higher age in years [Age↑ (years)] per SD (13.9 years), and the interaction of higher CAG triplet repeats with higher age (CAG × Age) as influencing variables. The symbols and error bars depict the mean odds ratios and 95% confidence intervals, which are also given right next to the symbols. **(B)** Frequency of PwHD without AID (No AID, gray) as well as PwHD with an AID of the five categories [blue: arthropathy-dominant AID of musculoskeletal and connective tissues (arthropathic AID, Arthro), light yellow: endocrine AID (Endo), orange: dermatological AID (Derm), light blue: gastrointestinal AID (Gastro), light green: other AID (Other)] in control participants (CP, oblique stripes) or HD mutation carriers (people with the HD mutation, PwHD, no stripes) categorized by length of the pathological CAG triplet expansion as indicated. The CAG size of the CAG triplet expansion length-defined groups as well as the group sizes is indicated. **(C)** Frequency of the four AID categories (AID_cat_)—arthropathic AID (blue), endocrine AID (light yellow), dermatological AID (orange), and gastrointestinal AID (light blue)—among PwHD when categorized as those with 36–40, 41–43, and >43 CAG triplet repeats in the pathological Huntingtin allele. **(D)** Forest plot showing the odds ratios of a higher CAG repeat length (CAG↑) per CAG, higher total functional capacity (TFC↑), female sex, and higher age (Age↑) per year in a PwHD core group with Shoulson and Fahn stage 1 and 2 with 41–44 CAG repeats and age from 36 to 72 years. Statistical analysis was performed using the Mann–Whitney *U* test [**(A)**, upper panel] and MLRA [**(A)**, lower panel] with the identification of significant interactions between independent variables and dichotomous variables being effect-coded as −1 and 1 and continuous variables being *z*-standardized; **(B)** five separate chi-square tests (*χ*^2^) comparing the number of participants with an AID to those without in each CAG group (upper panel) or comparing the number of those with an AID of the four major AID categories (the four lower panels) to those without such an AID. Each chi-square test was followed by seven pairwise Bonferroni-corrected Fisher’s exact tests, each comparing one CAG repeat group to CPs; and **(C)** four Fisher’s exact tests. **(D)** In the core group, the MLRA was performed without *z*-standardization, as the continuous variables CAG and FTC were not normally distributed; n.s. = not significant, *p < 0.05, **p < 0.01, ***p < 0.001, ****p < 0.0001.

One of the most prominent characteristics of the HD mutation is that individuals with HD carry a specific number of excess CAG triplets above 35 CAGs that are largely stable during their lifetime in white blood cells, while this number might increase considerably in other tissues, especially nerve cells, due to somatic instability, and both influence disease onset and, if very high, disease phenotype (1, 23). To test whether the exact number of excess CAG repeats has an impact on the likelihood of an AID in PwHD, the CAG triplet repeat lengths of the pathological alleles in PwHD with at least one AID were compared to those with and without an AID. CAG repeat length proved to be lower in the 709 AID-positive PwHD [median (IQR): 42 (41–44)] than in the 9,885 AID-negative PwHD [43 (41–45), Mann–Whitney *U* test, *p* < 0.0001; **Figure 3A**, upper panel]. MLRA among all PwHD confirmed that higher pathological CAG numbers were associated with a reduced probability of an AID as comorbidity documented at enrolment [OR (95% CI) per SD (9.9 repeats) higher CAG: 0.69 (0.61–0.78), *p* < 0.001; **Figure 3A**, lower panel]. In contrast, among the CPs, the upper normal CAG triplet repeat length was irrelevant regarding the AID frequency (**Supplementary Figure 12A**). When analyzing which of the AID subgroups contributed to this CAG dependency, it became evident that this was largely due to arthropathic AIDs, which in the low-CAG PwHD group (36–40 CAG) were documented in 3.4% (51/1,493), 1.7-fold more often than in CPs (Fisher’s exact test, Bonferroni-corrected, *p* = 0.045), while arthropathic AIDs were documented 3.9-fold less often in PwHD with >46 CAD repeats compared to CPs (8/1,570, 0.5%, Fisher’s exact test, Bonferroni-corrected, *p*<0.001; **Figure 3B**). The cohort with endocrine AIDs showed a similar but less pronounced trend for a lower frequency at higher CAG repeats. No such associations were found among CPs when grouped according to the larger *HTT* allele with normal CAG repeat length (**Supplementary Figure 12B**). When PwHD were grouped into three groups with low (36–40; *N* = 1,493), intermediate (41–43; *N* = 4,866), and high (>43; *N* = 4,235) CAG repeats (**Figure 3C**), the percentage of arthropathic and less so of endocrine AIDs declined with increasing CAG repeat length. Of note, within the Enroll-HD PDS, arthropathic AIDs were often documented using the largely unspecific code “unspecified arthritis” (107/199, 53%), followed by RA itself (40/199, 20%), then ankylosing spondylitis (17/199, 9%), and other rarer diagnoses (**Supplementary Figure 13A**). However, as the proportion of more unspecific diagnoses was not higher in CPs compared to PwHD, we did not find any evidence of a coding bias (**Supplementary Figure 13B**). Interestingly, the frequencies of participants documented with either the common specific arthropathic AID diagnoses of RA or ankylosing spondylitis declined among all PwHD with increasing CAG repeat numbers (1.8-fold in 41–43 CAG and 4.4-fold in >43 CAG compared to 36–40 CAG, Fisher’s exact test, *p* = 0.002), quite similar to the most common unspecific diagnosis “unspecified arthritis” (1.3-fold in 41–43 CAG and 5.0-fold in >43 CAG compared to 36–40 CAG, Fisher’s exact test, *p* < 0.0001; **Supplementary Figures 13C,D**). In CPs, the frequency of an arthropathic AID being documented increased with age at enrolment [Spearman *r* (95% CI) = 0.12 (0.08–0.16), *p* < 0.0001; **Supplementary Figure 14A**]. In contrast, age at enrolment strongly dropped in PwHD with increasing CAG repeat numbers [Pearson *r* (95% CI) = −0.52 (−0.53 to −0.50), *p* < 0.0001; **Supplementary Figure 14B**]. Due to these strong biases and low numbers of PwHD with high CAG repeats, we defined a PwHD core cohort restricted to the four most common CAG repeat lengths (41 to 44, **Supplementary Figure 14C**), early HD (stages 1 and 2, **Supplementary Figure 14D**) with age at enrolment within the 2.5th (36 years) to the 97.5th (72 years) percentile of PwHD with 41–44 CAG repeats and stage 1 or 2 at enrolment (**Supplementary Figure 14E**). This PwHD core cohort comprised 86 participants with an arthropathic AID documented (among those, 19 with RA) and 3,337 without an arthropathic AID. Still, within this subcohort, those with an arthropathic AID had a slightly and significantly higher age at enrolment [AID_artho_ vs. no AID_arthro_, median (IQR): 58 (53–64) years vs. 54 (49–61) years, Mann–Whitney *U* test, *p* < 0.0001] and had slightly lower CAG repeats [42 (41–42) vs. 42 (42–43), Mann–Whitney *U* test, *p* < 0.0001] but also slightly lower total functional capacity (TFC) scores [10 (8–12) vs. 11 (9–13), Mann–Whitney *U* test, *p* = 0.003; **Supplementary Figure 15A**]. Upon univariate analysis, with each additional CAG, the percentage of participants with an arthropathic AID dropped on average (mean ± SD) by 45% ± 13 % (Fisher’s exact test, *p* < 0.0001; **Supplementary Figure 15B**), as did the percentage of PwHD documented with RA (−56% ± 22% each higher CAG, **Supplementary Figure 15C**), although due to the 4.5-fold lower frequency of RA vs. all AID_arthro_ (*N* = 19 vs. *N* = 86), this decrease failed to be statistically significant (Fisher’s exact test, *p* = 0.139). Upon MLRA taking age at enrolment, sex, and TFC into account, each higher CAG was associated with a decreased risk for an arthropathic AID as indicated by a mean odds ratio of 0.57 (95% CI: 0.44–0.74, *p* < 0.0001; **Figure 3D**). Although the number of participants with RA was low for a model with multiple variables, an MRLA to explore the CAG dependency of RA resulted in a mean odds ratio of 0.53 (95% CI: 0.30–0.90, *p* = 0.018) per higher CAG (**Supplementary Figure 16A**). Of note, MLRA with a dichotomized dataset (≤41 vs. >41) with age and sex as influencing variables confirmed an increased risk for PwHD with low CAG repeats being documented with an arthropathic AID [OR (95% CI): 1.25 (1.03–1.53), *p* = 0.027], while there was an opposite trend at high CAG repeats [OR (95% CI): 0.85 (0.72–1.02), *p* = 0.08; **Supplementary Figure 16B**]. In summary, the specific number of excess CAG triplets in the mutant *HTT* allele had differential effects on the frequency of arthropathic AIDs as a secondary diagnosis in PwHD with a low pathological CAG number representing a risk factor, while a high excess number of CAG repeats tended to have an opposite effect.

## 4 Discussion

Analyses of brain tissue and blood biomarkers as well as *in vitro* studies with cells of the innate immune system indicate immune activation not only in the HD brain but also systemically (3, 8–10). Activation of innate immunity is a prerequisite for the initiation of both cellular and antibody-mediated adaptive immunity (16). Thus, we hypothesized that breaches of self-tolerance might occur more frequently in the presence of HD-associated immune activation. We tested this hypothesis by asking whether there is a higher prevalence of diverse AIDs documented as comorbidities in HD mutation-positive compared to HD mutation-negative participants of the Enroll-HD observational study (20).

Of note, our results were not as clear-cut as expected. In general, AIDs were documented in PwHD at a frequency not different from that of CPs. However, the distribution of AID subgroups among PwHD differed substantially when compared to the control population. Thus, our results suggest that the HD mutation indeed, but differentially, modifies the risk for specific AIDs. Endocrine AIDs were documented much less frequently in PwHD than in CPs, primarily due to the rarity of HT when the HD mutation was present. The tendency of dermatological AIDs being more frequent in PwHD could be explained by a higher proportion of PwHD documented with psoriasis as a comorbidity. Finally, we could demonstrate that the length of the pathological CAG triplet expansion differentially affects the AID frequency in PwHD, an effect largely explained by AIDs associated with arthritis being more frequent at the low pathological range while becoming rarer with increasing excess CAG repeat numbers. Of note, our results reached statistical significance even when prominent confounders—sex and age—that influence the likelihood of an AID diagnosis were accounted for.

Of course, our analysis has several limitations that need to be considered carefully when interpreting our results. Importantly, in the Enroll-HD electronic case report form, comorbidities are entered by site personnel in text format first and are later centrally converted to an ICD-10 code lacking the last of maximal two digits after the full stop. During these procedures, there is the possibility that the text entry describes the pathologic state resulting from an AID, e.g., hypothyroidism instead of HT, leading to an ICD-10 code not representing an AID. In addition, as usually associated with the departments of neurology, or less frequently, psychiatry, the site personnel’s limited knowledge of non-neurological, e.g., rheumatological, AIDs might have led to rather unspecific AID diagnoses, i.e., “unspecified arthritis” in arthropathic AIDs. Finally, CPs, usually family members, do not have medical records on file at the respective study center, while most of the HD mutation carriers do. In European Enroll-HD sites, medical files are the source material for on-site monitoring.

We could show that AIDs potentially missed due to truncation of the ICD code are very few (*N* = 3). In addition, we did not find any evidence for relevant biases between CPs and PwHD, neither regarding coding of hypothyroidism instead of HT nor for more frequent unspecific arthropathic AID codes. Of note, if relevant, this bias should decrease AID diagnoses in the CP compared to the PwHD cohort, thus leading to a comparatively more AID-positive PwHD, e.g., as found for psoriasis. Indeed, the documented frequency of psoriasis among European Enroll-HD CPs (0.8%), but not the PwHD (1.5%), was slightly below the estimated prevalence in Western Europe (2.1%, 95% CI: 1.1–4.1%) (24). Thus, it cannot be excluded that our finding that psoriasis is more prevalent in PwHD might result from a documentation bias. However, the reverse must be applied to HT, which then should be even less frequent in the PwHD compared to the non-HD population as compared to the documentation in the Enroll-HD PDS. Still, when compared to the estimated HT prevalence in Europe [8.0% (95% CI: 5.2–11.4%)] (25), its frequency among both PwHD (0.5%) and Enroll-HD CPs (1.2%) was considerably lower. We argue that this might be explained by the fact that HT might indeed be often coded as hypothyroidism, which was coded almost 4-fold more often than HT. In contrast to psoriasis and HT, RA, which is characterized by a rather consistent prevalence of 0.5% to 1.0% worldwide (26), was documented within the range of the expected frequency in Enroll-HD CPs (0.5%), even more so when considering that many RA patients might have been coded as “unspecified arthritis”. For the frequency of RA and related disorders, it may be argued that the inverse association with the number of excess CAG triplet repeats is confounded by the fact that the onset of RA usually peaks in the third to fifth decades of life (26). HD patients with large CAG triplet repeat expansions at risk of developing RA might not live until the age when RA would manifest or at that point might be too impaired to be enrolled in Enroll-HD. However, our results turned out to be robust even when the range for CAG repeat expansion was restricted to 41 to 44 CAGs, stage at enrollment to early manifest HD, and subsequent adjustment for age at enrollment and functional capacity.

Even when considering the limitations of our approach, it seems reasonable to assume that the HD mutation indeed disease-specifically affects the likelihood of a carrier to develop certain AIDs, especially a generally reduced risk for HT, a CAG repeat-length-dependent risk modification of the arthropathic AIDs including RA and, with less certainty, an increased risk for psoriasis. Thus, it can be asked whether inflammatory changes associated with the pathogenesis of these AIDs match changes found in HD in AIDs with where HD increases the risk or the opposite when carriership of the HD mutation is associated with a decreased risk. In psoriasis, which is characterized by the development of erythemato-squamous plaques on the patient’s skin, activated dermal dendritic cells attract Th17 cells, recognizing epidermal autoantigens, e.g., keratin-7 (27). It is assumed that interleukin-23 and TNF-α produced by dendritic cells drive the activation and differentiation of interleukin 17-producing effector T helper cells (Th17 cells), which play a crucial role in psoriatic plaque formation (28–30). Interestingly, not only elevated blood TNF-α (9) and IL-23 (31) levels in HD but also increased TH17.1 cells in HD CSF have been reported, including IL-17 (4). Although it is currently unknown how the HD mutation increases TNF-α and IL-23 levels, overabundance of these cytokines and IL-23-stimulated differentiation of TH17 cells might well lower the threshold for overt psoriasis in genetically susceptible individuals. Thus, all this could be interpreted as supporting evidence for our finding that PwHD might have a higher risk for psoriasis. In HT, lymphocytic infiltration of the thyroid gland is associated with follicular cell destruction, subsequent fibrosis, and often hypothyroidism (32). It is characterized by the presence of antithyroid antibodies, especially against thyroglobulin (TG) and thyroid peroxidase (TPO) (32). Intriguingly, increased production of TNF-α and IL-23, as well as IL-17, cytokines reportedly involved in psoriasis, are also thought to be relevant for the immune pathology of HT (33–36), which, according to our analysis and in contrast to psoriasis, is less frequent in PwHD. Last but not least, RA is a systemic AID characterized by inflammatory synovitis and arthritis, typically involving the small joints of the hands and feet, which—if seropositive—is marked by the presence of autoantibodies to post-translationally modified proteins, e.g., citrullinated, carbamylated, or modified by malondialdehyde adducts, and less specific autoantibodies, known as rheumatoid factors, which bind the Fc portion of immunoglobulins (26). In contrast to psoriasis and HT, in RA, multiple potential paths lead to a common clinical presentation (26). Thus, the interaction of the HD mutation with the risk for RA is probably as complex as the pathogenesis of RA. Finally, indirect effects of the HD mutation have to be considered. Notably, the gut–immune axis is thought to modulate the risk for autoimmunity (37). As the gut microbiome has been reported to be altered in PwHD (38), gut dysbiosis might explain parts of our findings. Taken together, it can be assumed that the HD mutation modulates AID risk via a plethora of underlying mechanisms.

### 4.1 Conclusion

In conclusion, our findings based on the documentation of comorbidities within the Enroll-HD PDS are in line with the hypothesis that carriership of the *HTT*-CAG triplet repeat expansion modifies the development of different AIDs in a very complex fashion. A pathological *HTT*-CAG triplet expansion might increase the risk for developing psoriasis, is likely to be generally protective against HT, and might modify the risk for RA and other arthropathic AIDs in a CAG-length-dependent manner. However, due to the nature of our study, our findings must be interpreted as merely hypothesis-generating. They warrant verification using independent datasets like national registries (39) or testing the prevalence of disease-specific antibodies, like anti-TG or anti-TPO IgG as markers for HT (32) or anti-citrullinated peptide antibodies or rheumatoid factor for RA (26) in Enroll-HD biosamples.

## Data Availability

The original contributions presented in the study are included in the article/**Supplementary Material**. Further inquiries can be directed to the corresponding author. The current Enroll-HD PDS (https://enroll-hd.org/for-researchers/datasets/#hd-datasets/) can be obtained from CHDI upon application.
