# Supplementary material for: The pathological Huntingtin CAG triplet expansion differentially affects the diagnosis of systemic and organ-specific autoimmune diseases
Source: Front Immunol. 2026 May 8;17:1689962. doi: 10.3389/fimmu.2026.1689962 (PMC13194399; doi:10.3389/fimmu.2026.1689962)
Supplement: Supplementary file 1 [file Table1.docx]

**The pathological Huntingtin CAG triplet expansion differentially affects the diagnosis of systemic and organ specific autoimmune diseases – Supplementary Material**

| **ICD code with one digit after full stop** | **Name** | **2nd digit after full top** | **Name** | **Definite**  **AID** |
| --- | --- | --- | --- | --- |
| D68.3- | Haemorrhagic disorder due to intrinsic circulating anticoagulants, antibodies, or inhibitors | **1** | **Haemorrhagic disorder due to antibodies against factor VIII (Acquired Hemophilia A)** | **yes** |
|  |  | **2** | **Haemorrhagic disorder due to antibodies of coagulation factors (IXa, Xa, XIa, vWF)** | **yes** |
|  |  | 3 | Haemorrhagic disorder due to coumarins (vitamin K antagonists) | no |
|  |  | 4 | Haemorrhagic disorder due to heparins | no |
|  |  | 5 | Haemorrhagic disorder due to other anticoagulants | no |
|  |  | **8** | **Haemorrhagic disorder due to other antibodies** | **yes** |
| D69.5- | Secondary thrombocytopenia | 2 | Heparin-induced thrombocytopenia Type I | no |
|  |  | **3** | **Heparin-induced thrombocytopenia Type II** | **yes** |
|  |  | 7 | Other secondary thrombocytopenia identified as refractory to transfusion | no |
|  |  | 8 | Other secondary thrombocytopenia not identified as refractory to transfusion | no |
|  |  | 9 | Other secondary thrombocytopenia, not otherwise specified | no |
| K83.0- | Cholangitis | **0** | **Primary sclerosing cholangitis** | **yes** |
|  |  | 1 | Secondary sclerosing cholangitis | no |
|  |  | 2 | Other cholangitis | no |
|  |  | 3 | Unspecified cholangitis | no |
| K86.1- | Other chronic pancreatitis | **0** | **Autoimmune pancreatitis** | **yes** |
|  |  | 1 | Hereditary pancreatitis | no |
|  |  | 8 | Other chronic pancreatitis | no |

**Supplementary Table 1: Definite autoimmune disease not encoded by an abbreviated ICD 10 with one digit after the full stop.** AID = autoimmune disease, vWF = von-Willbrand factor.


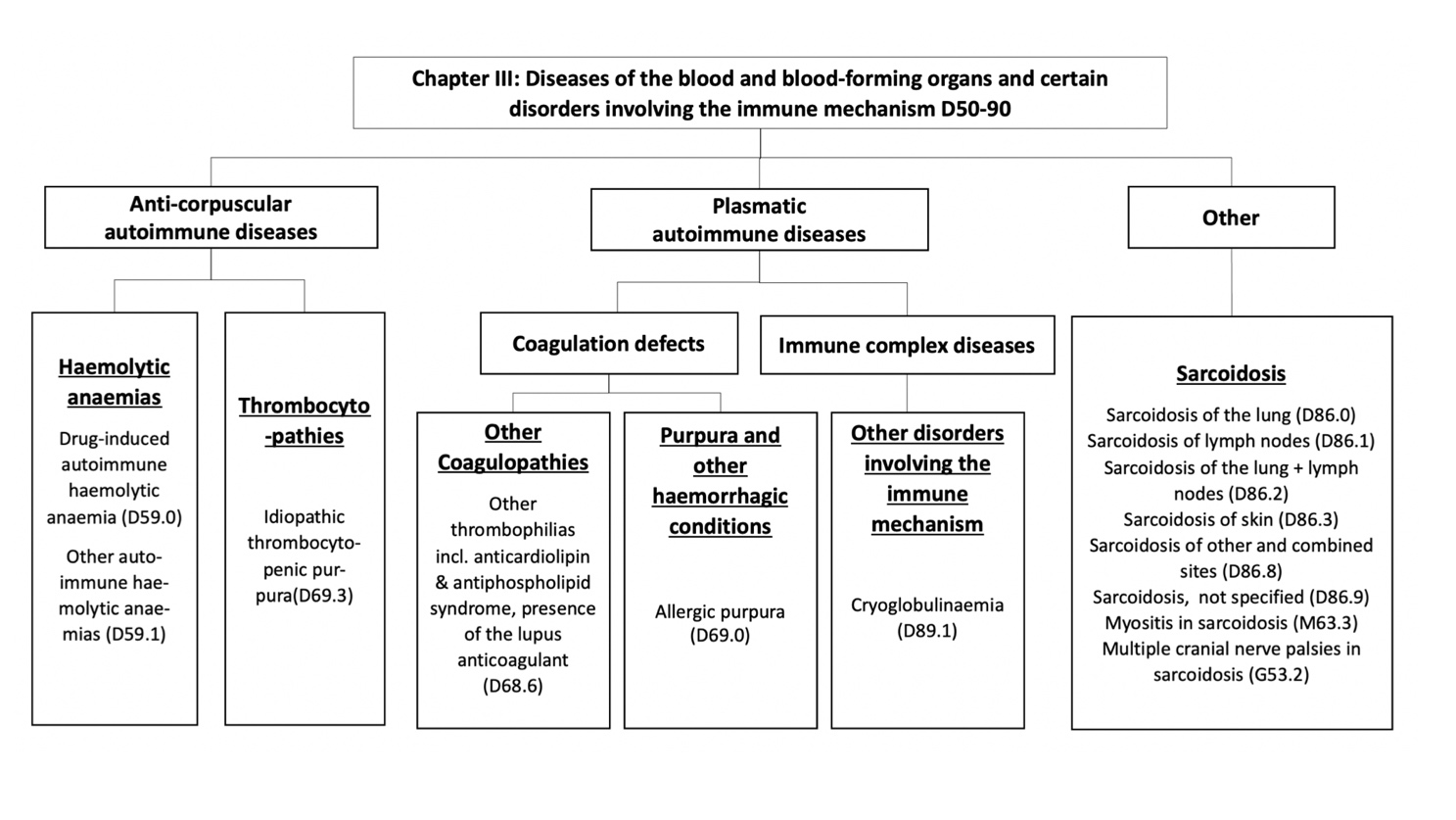


**Supplementary Figure 1: Codes from Chapter III of the ICD10 encoding definite autoimmune diseases in the Enroll-HD dataset.** Diseases definitely encoded as autoimmune diseases (AID) in codes until the penultimate digit following the full stop were grouped into AID with immune reaction against blood cells (anti-corpuscular), plasmatic and other AID. Myositis in sarcoidosis grouped in musculoskeletal AID (Chapter XIII, see Supplementary Figure 7) was grouped here.


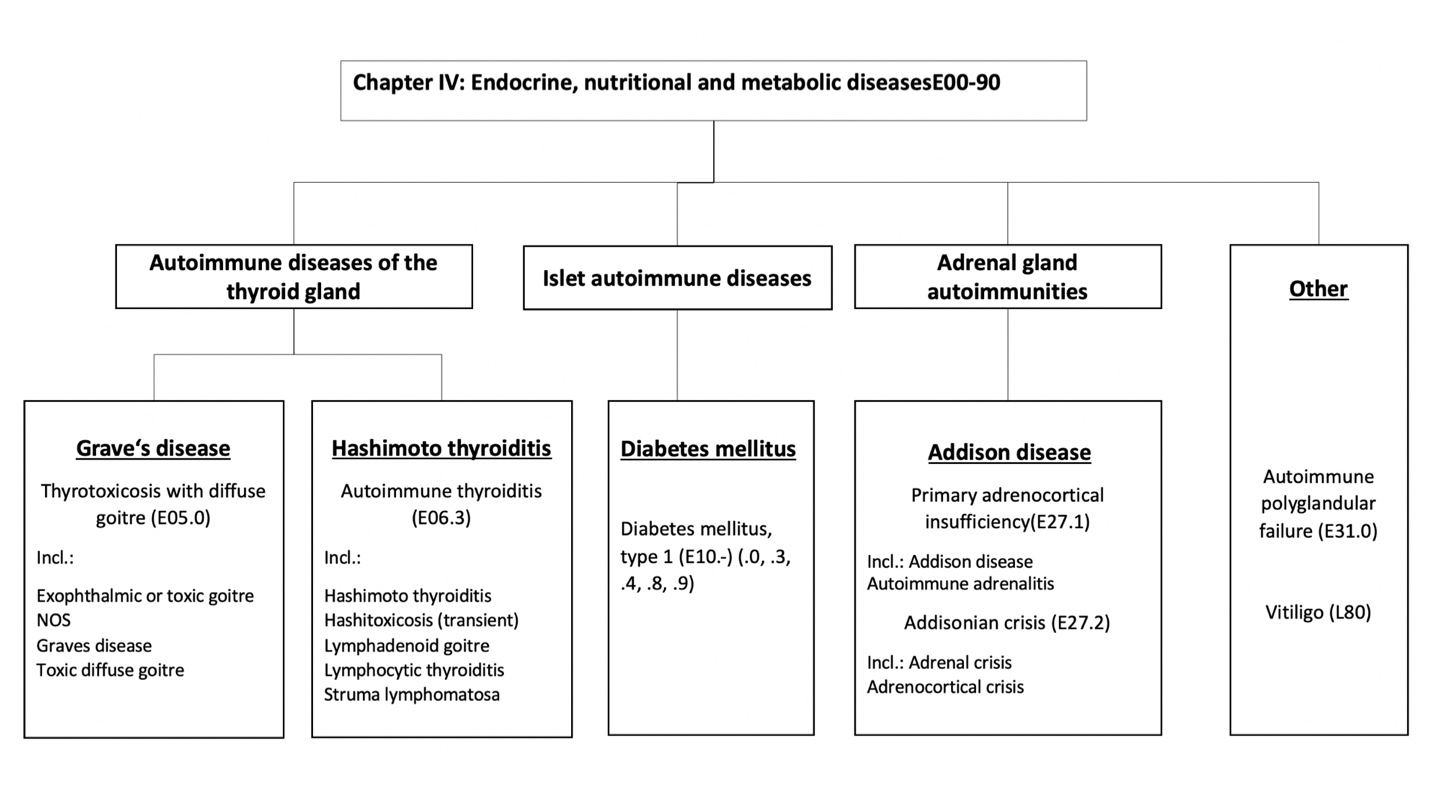


**Supplementary Figure 2: Codes from Chapter IV of the ICD10 encoding definite autoimmune diseases in the Enroll-HD dataset.** Diseases definitely encoded as autoimmune diseases (AID) in codes until the penultimate digit following the full stop were grouped into endocrine AID. Vitiligo usually grouped with dermatological AID (Chapter XII, see Supplementary Figure 6) was grouped here.


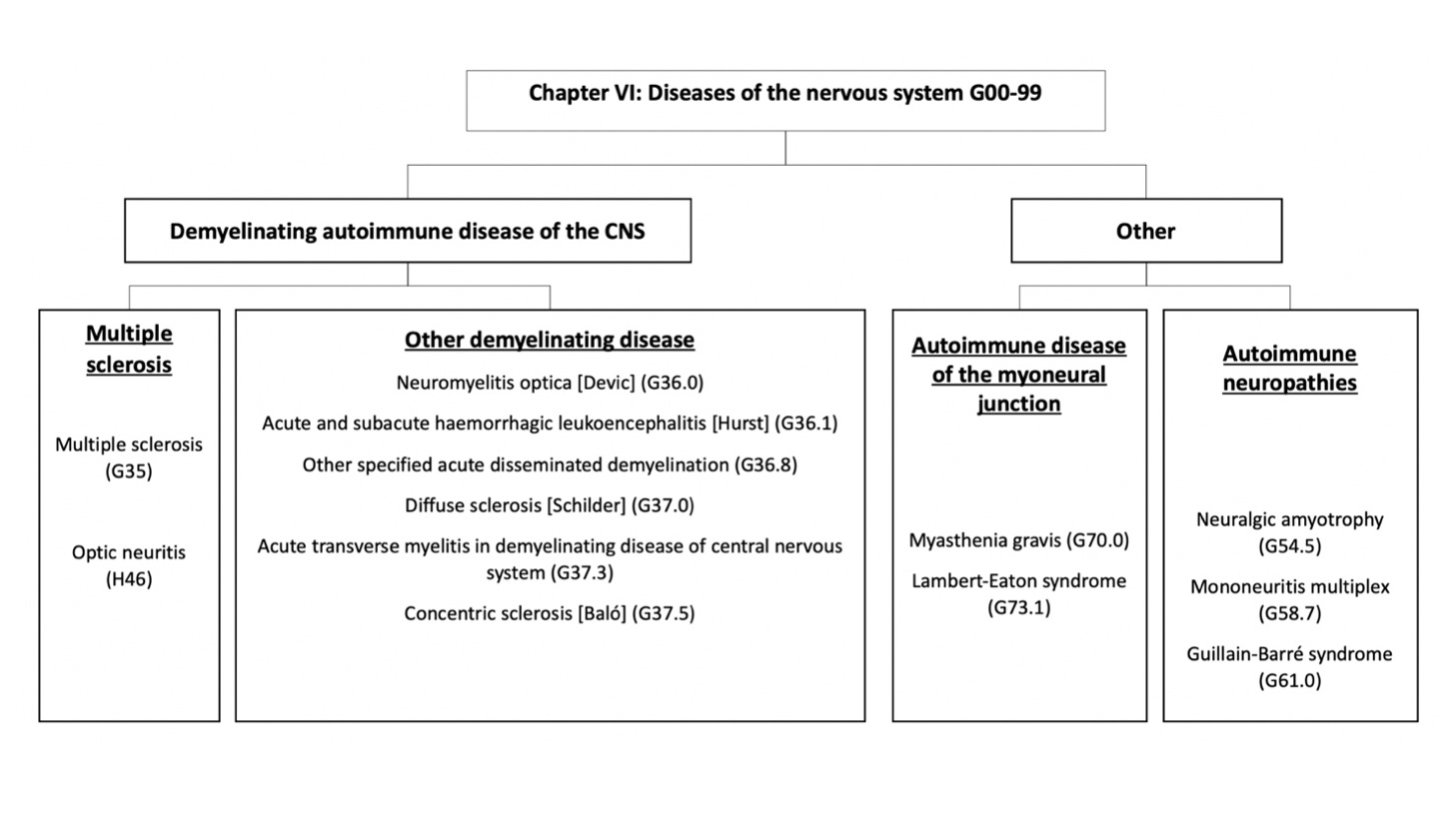


**Supplementary Figure 3: Codes from Chapter VI of the ICD10 encoding definite autoimmune diseases in the Enroll-HD dataset.** Diseases definitely encoded as autoimmune diseases (AID) in codes until the penultimate digit following the full stop were grouped as AID of the nervous system.


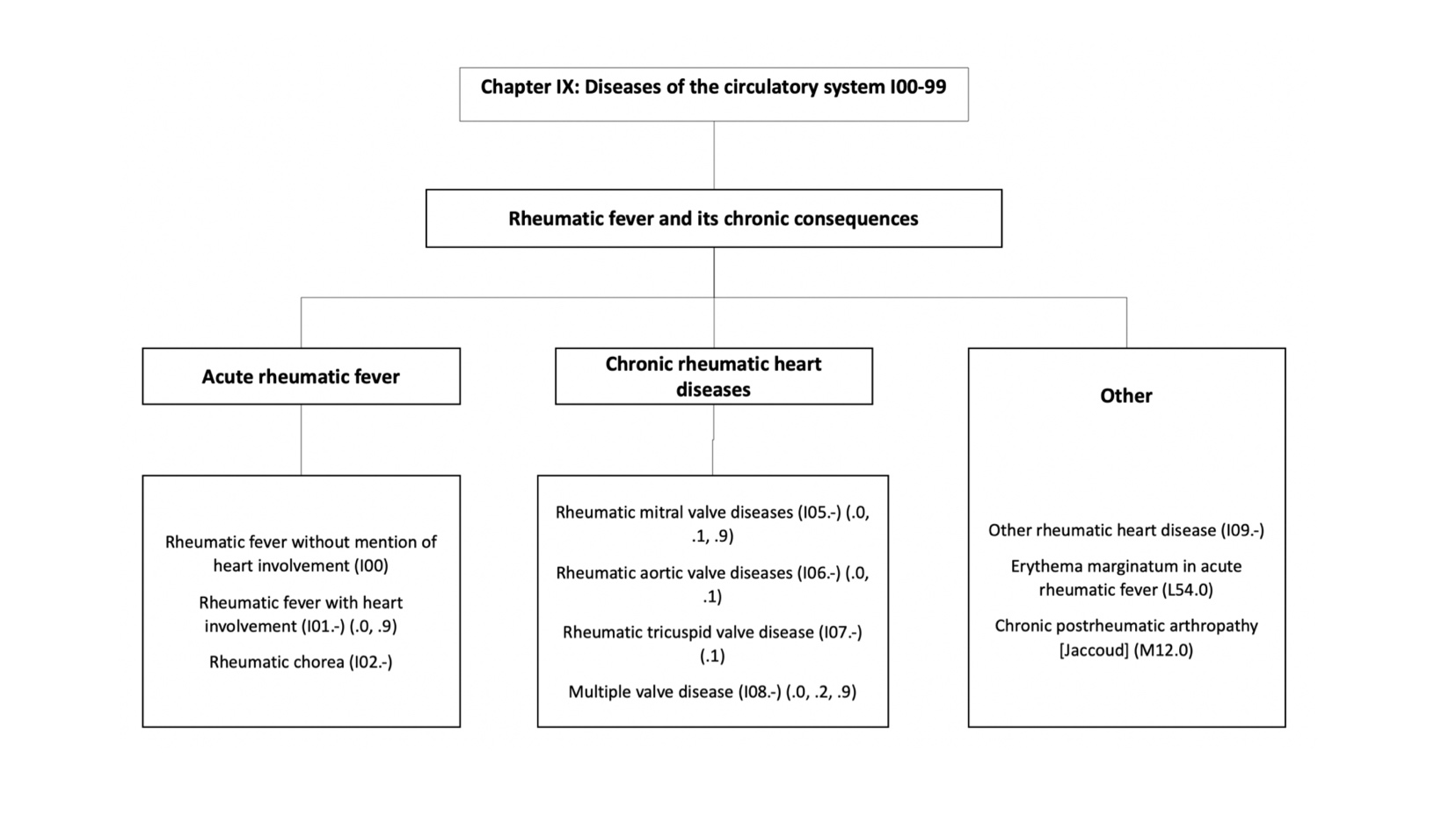


**Supplementary Figure 4: Codes from Chapter IX of the ICD10 encoding definite autoimmune diseases in the Enroll-HD dataset.** Diseases definitely encoded as autoimmune diseases (AID) in codes until the penultimate digit following the full stop were grouped as AID of the circulatory system.


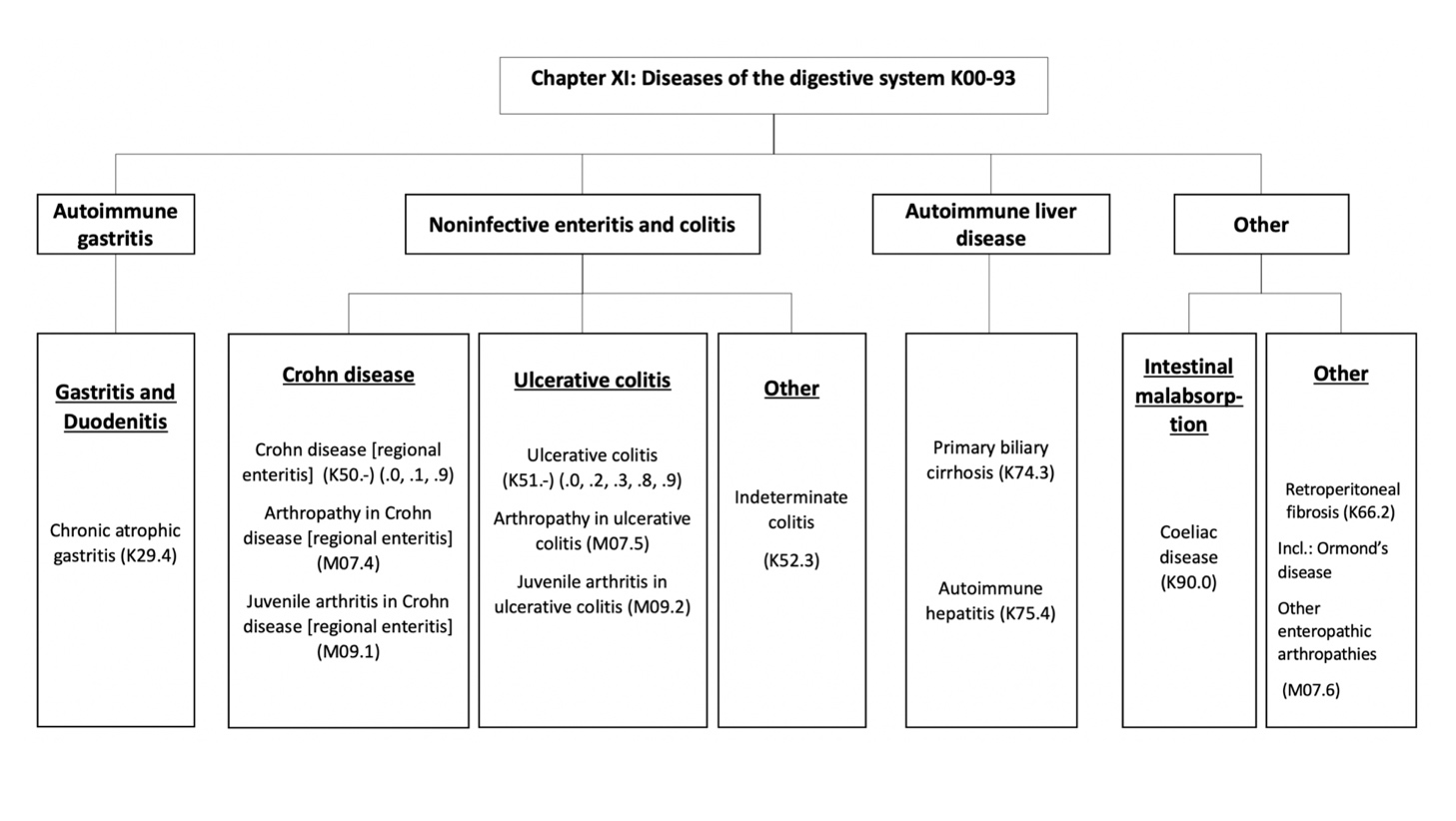


**Supplementary Figure 5: Codes from Chapter XI of the ICD10 encoding definite autoimmune diseases in the Enroll-HD dataset.** Diseases definitely encoded as autoimmune diseases (AID) in codes until the penultimate digit following the full stop were grouped as gastrointestinal AID.


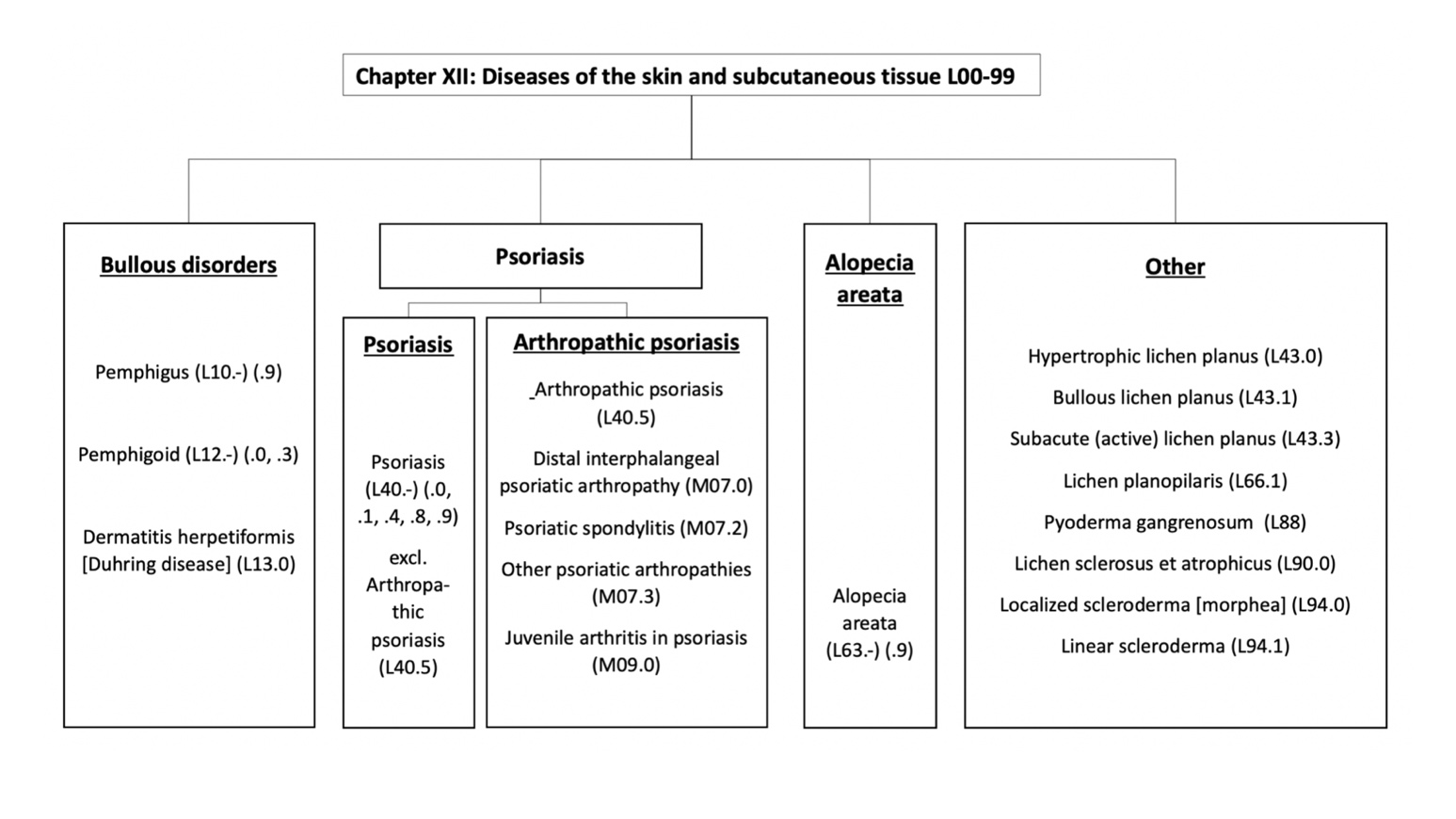


**Supplementary Figure 6: Codes from Chapter XII of the ICD10 encoding definite autoimmune diseases in the Enroll-HD dataset.** Diseases definitely encoded as autoimmune diseases (AID) in codes until the penultimate digit following the full stop were grouped as dermatological AID. Arthritis associated with psoriasis was also grouped here.


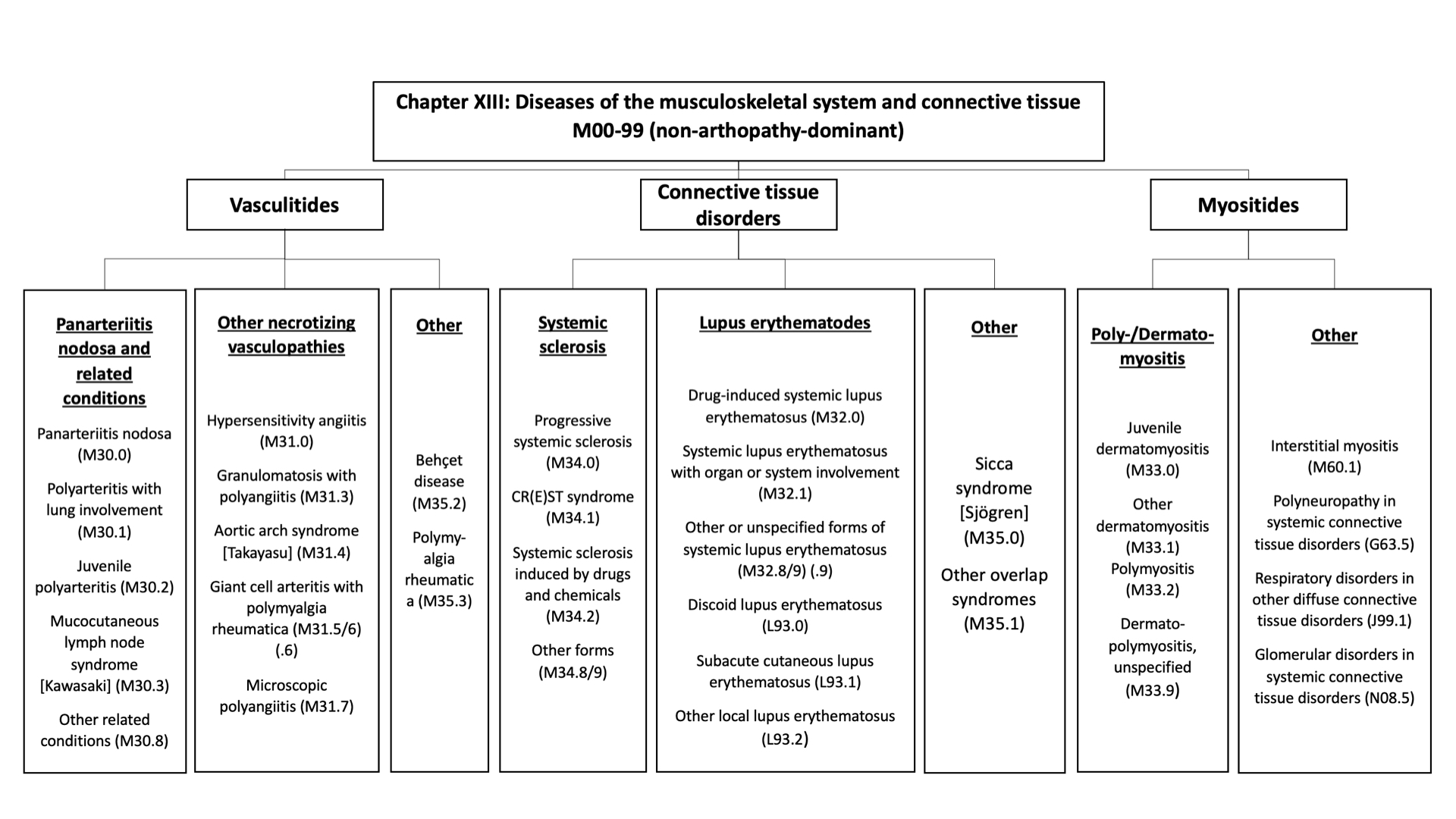


**Supplementary Figure 7: Codes from Chapter XIII of the ICD10 encoding definite autoimmune diseases in the Enroll-HD dataset typically not involving the joints.** Diseases definitely encoded as autoimmune diseases (AID) in codes until the penultimate digit following the full stop were grouped as usually non-artropathy-dominant musculoskeletal and connective tissue AID.


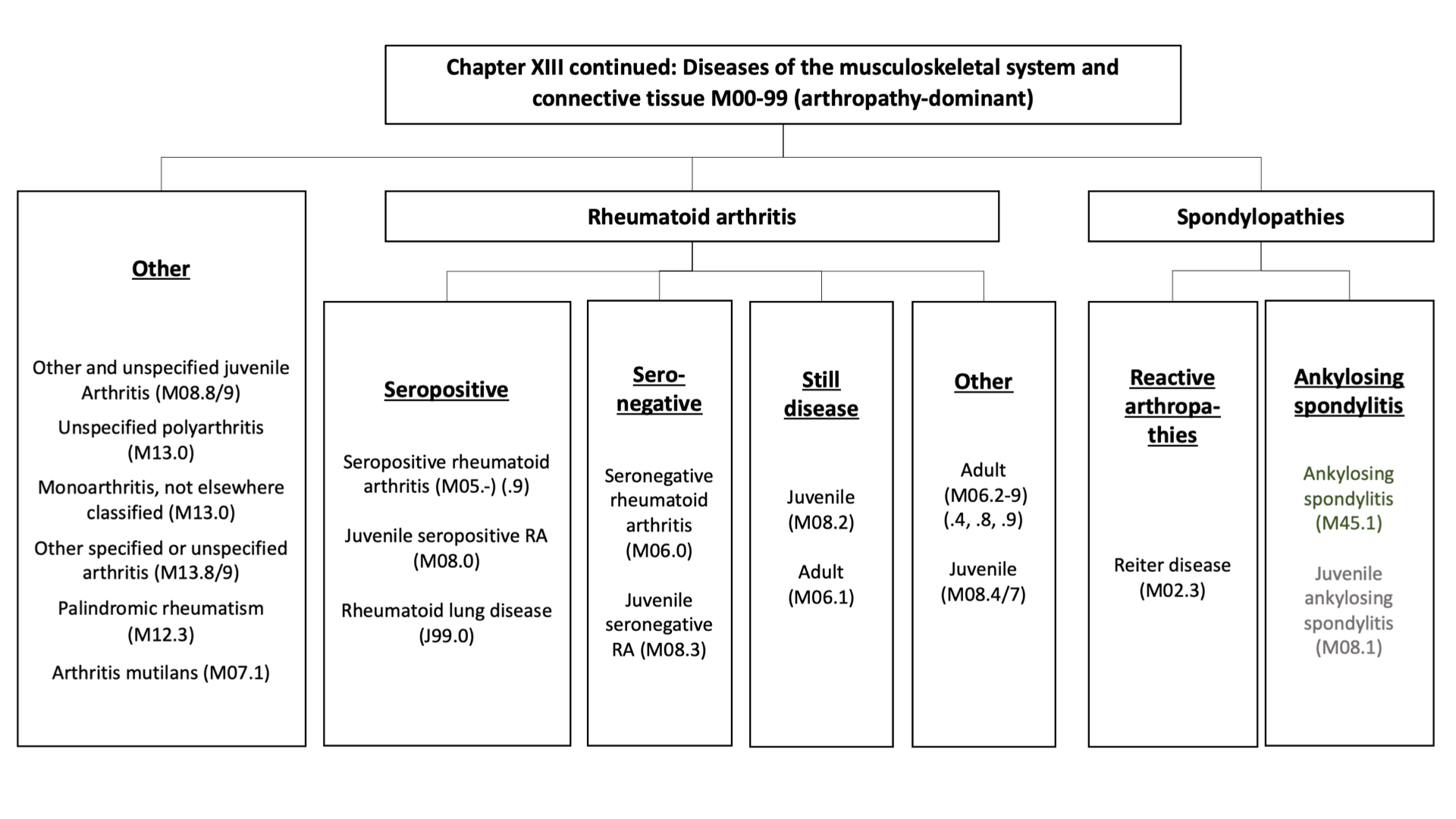


**Supplementary Figure 8: Codes from Chapter XIII of the ICD10 encoding definite autoimmune diseases in the Enroll-HD dataset typically involving the joints.** Diseases definitely encoded as autoimmune diseases (AID) in codes until the penultimate digit following the full stop were grouped as usually arthropathy-dominant AID of musculoskeletal and connective tissues.


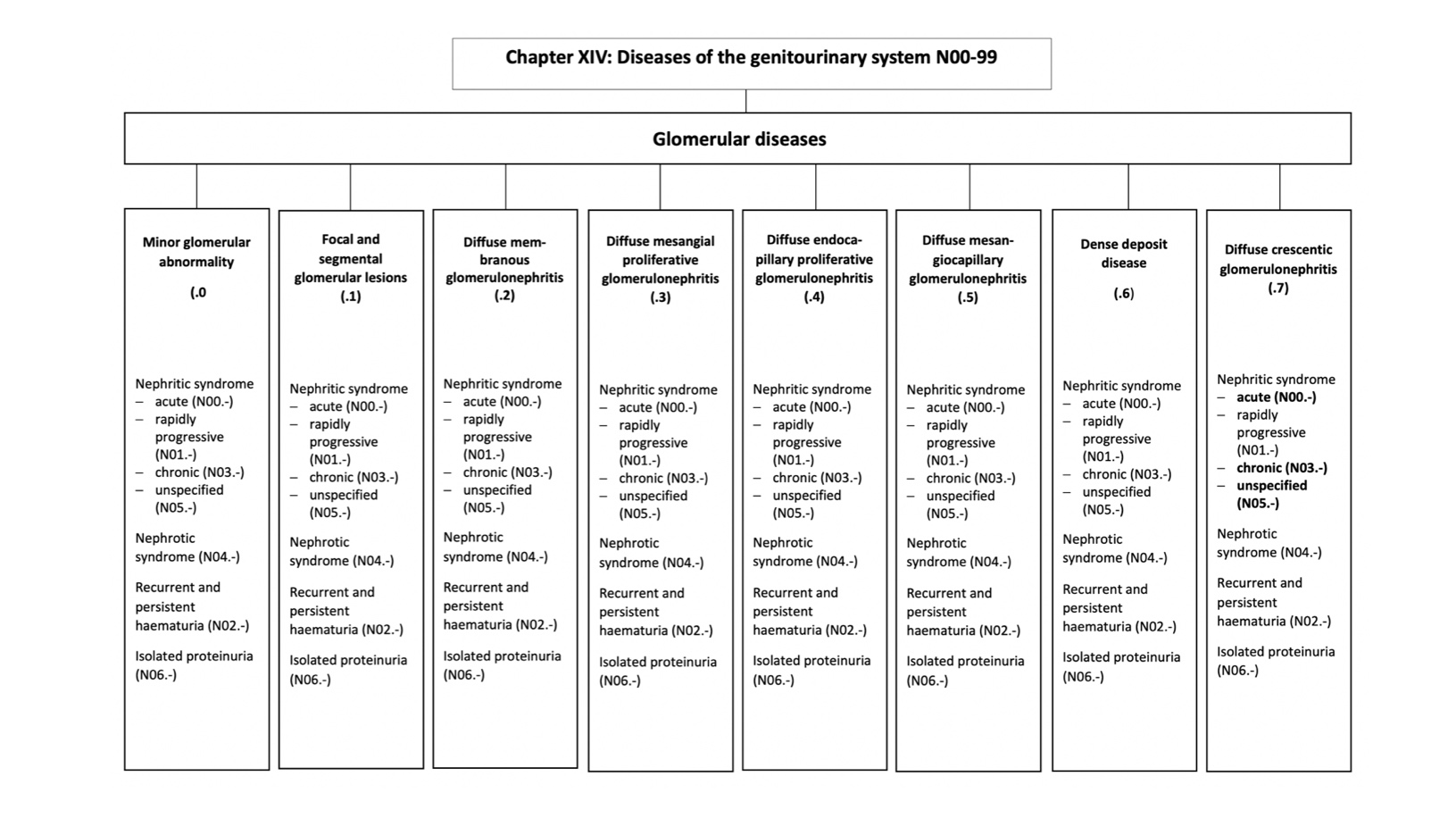


**Supplementary Figure 9: Codes from Chapter XIV of the ICD10 encoding definite autoimmune diseases in the Enroll-HD dataset typically.** Diseases definitely encoded as autoimmune diseases (AID) in codes until the penultimate digit following the full stop were grouped as AID of the genitourinary system.

| **Ambiguous ICD code** | **Name** | **PwHD**  **N= 10,594**  **(N, %)** | **CP**  **N=2,477**  **(N, %)** | **Total**  **N=13,071**  **(N/%)** |
| --- | --- | --- | --- | --- |
| D68.3 | Haemorrhagic disorder due to intrinsic circulating anticoagulants, antibodies, or inhibitors | 0 (0.00) | 0 (0.00) | 0 (0.00) |
| D69.5 | Secondary thrombocytopenia | 0 (0.00) | 0 (0.00) | 0 (0.00) |
| K83.0 | Cholangitis | 1 (0.01) | 0 (0.00) | 1 (0.01) |
| K86.1 | Other chronic pancreatitis | 1 (0.01) | 1 (0.04) | 2 (0.02) |

**Supplementary Table 2: Use of ICD10 codes turned ambiguous because of omission of the second digit following the full stop in the European Enroll-HD dataset**

**
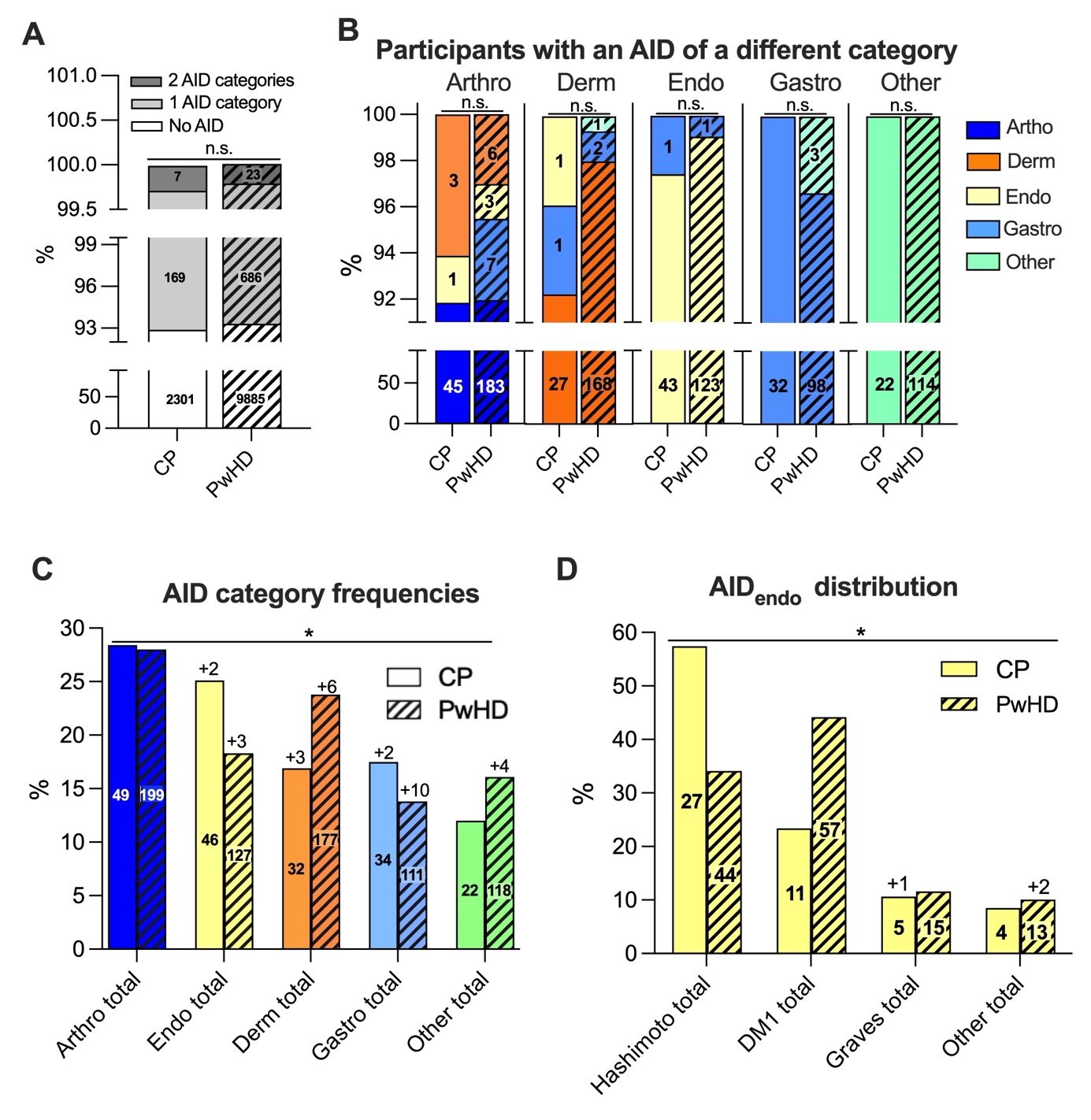
Supplementary Figure 10: No relevant effect of the elimination of double diagnoses on the differences in between-categories and within-category diagnosis distribution in people with the Huntington mutation and control participants** (A) Proportion of participants among control participants (CP, no pattern) and people with the Huntington mutation (PwHD, striped) with diagnoses of none of the five autoimmune disease (AID) categories (No AID), one AID category or two of the five AID categories “Arthropathy-dominant AID of the musculoskeletal and connective tissues” (Arthro), “Dermatological AIDs” (Derm), “Endocrine AID” (Endo), “Gastrointestinal AID” (Gastro) or “Other AID” (Other). (B) Relative percentage of participants with a diagnosis of an additional less frequent AID category that was deleted for the main analysis in Figure 1A shown separately for the five AID categories (Arthro: blue, Derm: orange, Endo: yellow, Gastro: light blue, Other: reseda). The percentage with participants without a diagnosis of an additional less frequent AID category is shown in the color of the respective main category. The percentage of participants with an AID of an additional category are shown in the color of that category in the same column. (C/D) The same analyses as Figure 1B and 2A but with the total number of all AID categories (C) or endocrine AID (D) coded in the study population. The number of diagnoses that were deleted due to the rule to assign each participant to the most frequent AID category (Figure 1B) or the most frequent endocrine AID (Figure 2A) only are shown above the columns. The numbers of participants (A/B), AID categories within the respective subgroup (C) or endocrine diagnoses (D) are given within the bar. Statistical analysis was performed using Fisher’s exact tests as indicated, n.s.= not significant, *p<0.05.

**
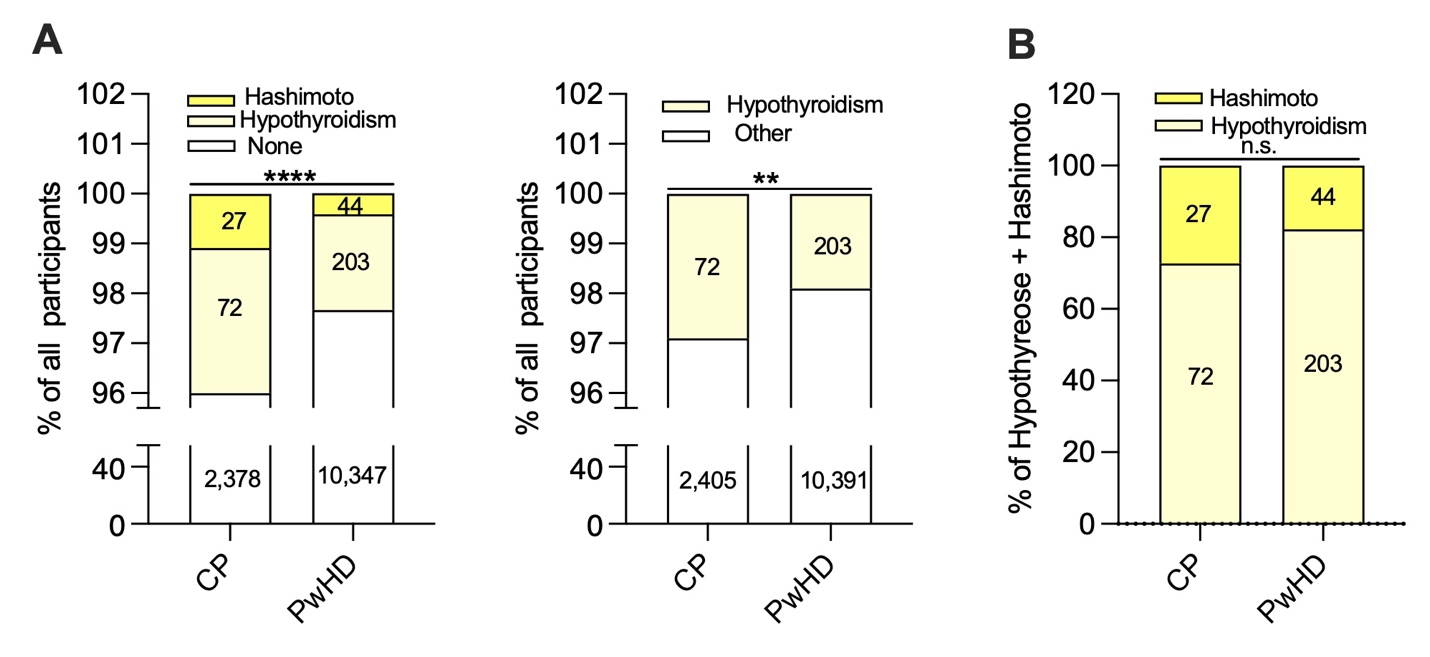
**

**Supplementary Figure 11: Assessment of potential coding biases in control participants compared to people with the HD mutation regarding Hashimoto thyroiditis and hypothyroidism. (**A, left panel) Frequency of the documentation of either Hashimoto thyroiditis (Hashimoto), its functional, unspecific outcome hypothyroidism without Hashimoto thyroiditis (Hypothyroidism) and neither of both (None) in control participants (CP) and people with the Huntington mutation (PwHD). (A, right panel) Frequency of hypothyroidism only compared no hypothyroidism (Other) in CP and PwHD. (B) Proportion of CP and PwHD diagnosed with either Hashimoto or hypothyroidism. The numbers of participants are given within the bars. The statistical comparisons were performed by Chi-square (A, left panel) or Fisher’s exact tests (A, right panel, B), n.s.: not significant, **p<0.01, ****p<0.0001.


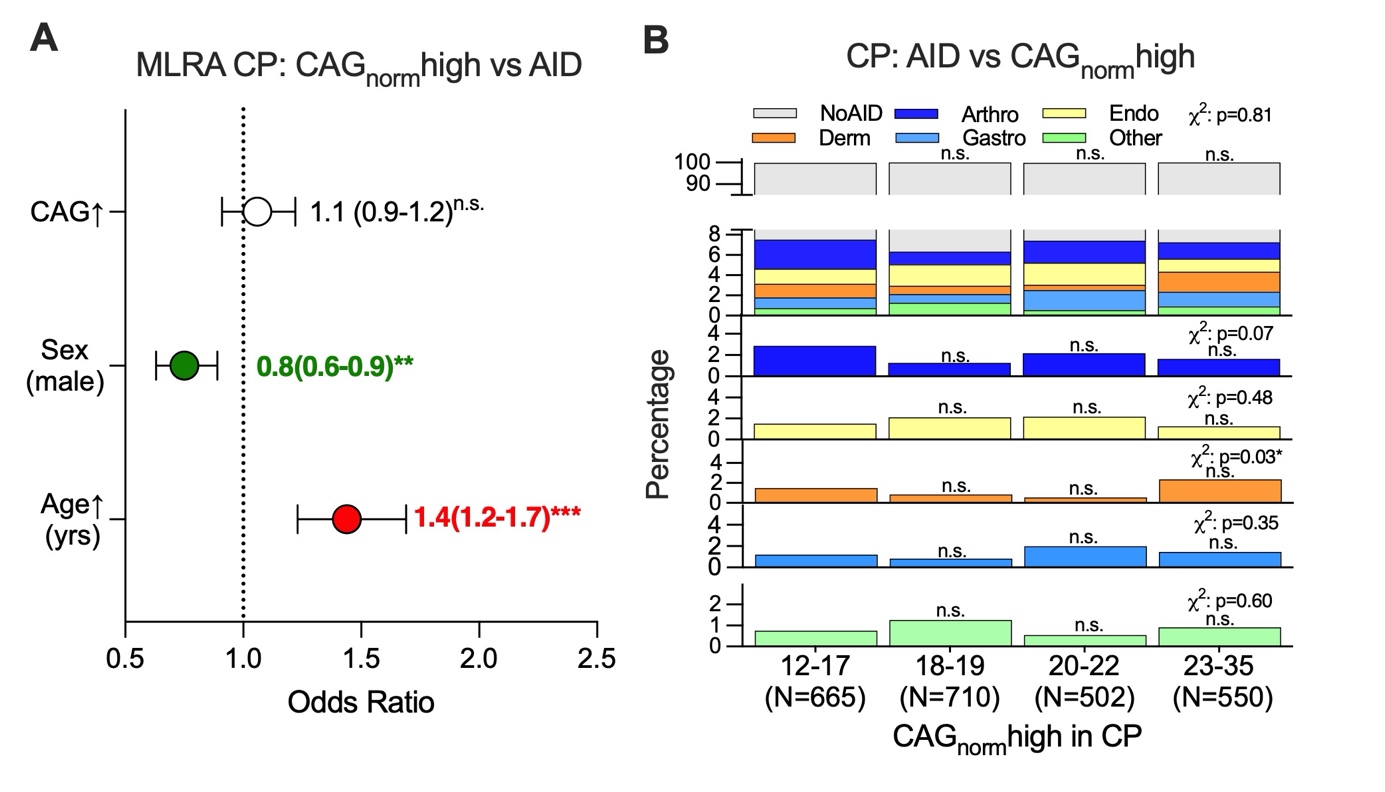


**Supplementary Figure 12: The higher CAG repeat number of both normal HTT alleles in control participants is not associated with relevant changes in the frequency of autoimmune diseases.** (A) Forest plot of the mean and 95% confidence intervals (95%CI) of the odds ratios (OR) for the diagnosis of an autoimmune disease (AID) in association with an increase of the higher of both normal CAG repeat lengths (CAG_norm_high) by one standard deviation (SD, CAG↑), male sex and an increase of age by one SD [Age↑(yrs)] in control participants (CP) obtained by multiple logistic regression analysis (MLRA). The means and 95%CIs of the ORs are given to the right of the error bars. An OR of 1 is indicated by the dotted line. Green: significantly lower than 1, red: significantly higher than 1. (B) Percentage of CPs with all AIDs together or all of those with one the main AID subgroups separately with respect to the higher normal CAG repeat length. The upper graph shows the percentage of CPs with AID with each CP assigned to the most frequent AID category if AIDs of two categories [arthropathy-dominant AID of musculoskeletal and connective tissues: Arthro (blue), endocrine AID: Endo (yellow), dermatological AID: Derm (orange), gastrointestinal AID: Gastro (light blue), other: Other (light green)] were documented as well as those without an AID (No AID, grey) as a stacked bar graph subgrouped according to CAG_norm_high as indicated. The number of CPs in each CAG subgroup is given below. The percentage of all CPs with each AID subgroup separately is depicted in the lower graphs. Statistical analysis was performed by (A) MLRA and (B) by Chi square tests (χ2) and pairwise Fisher’s exact tests compared to the group with the lowest normal CAG number. P values for the pairwise Fisher’s exact tests were Bonferroni-corrected; the p-values for the Chi square test are given within the graphs, n.s. = not significant, *p<0.05.


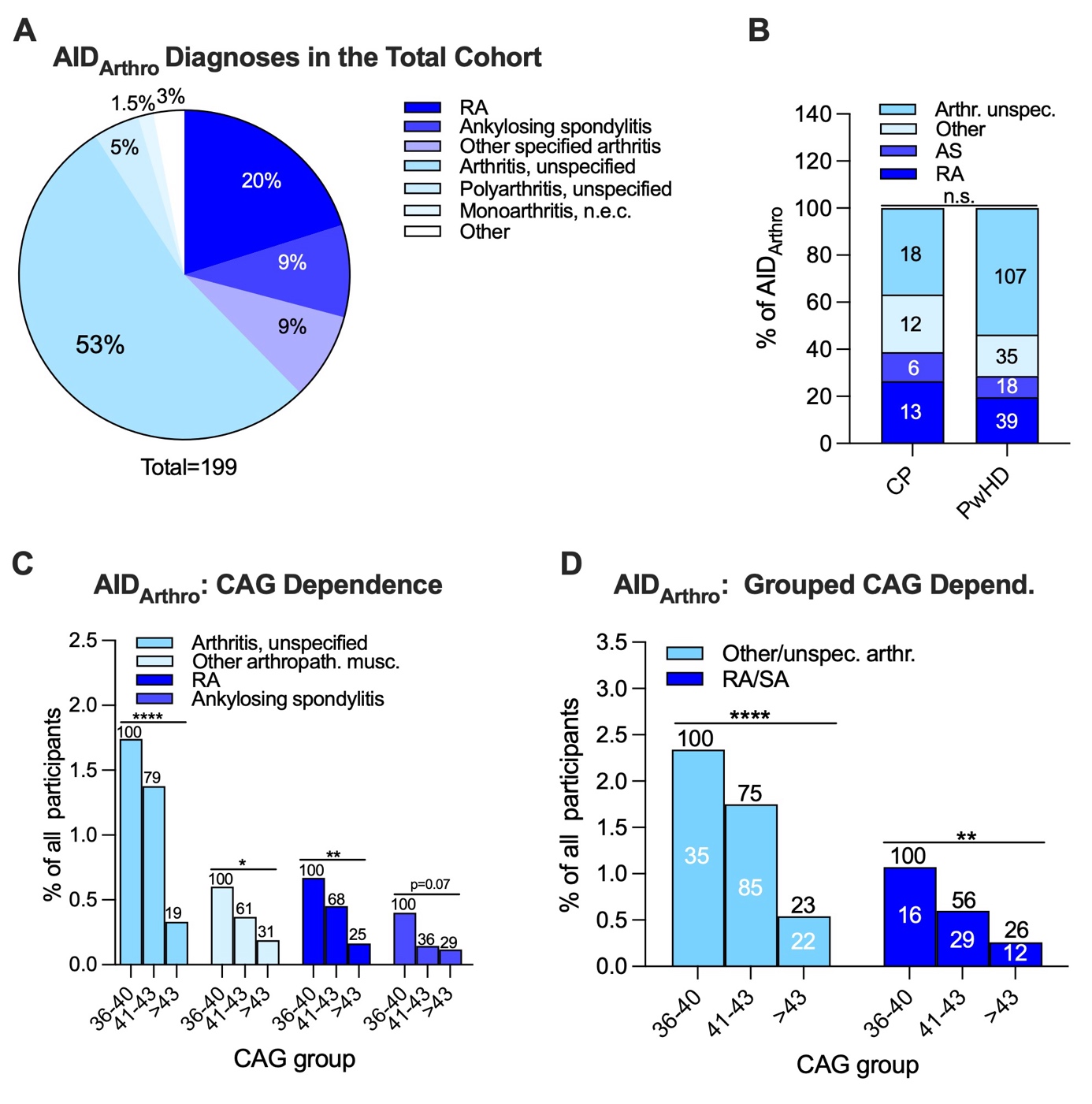


**Supplementary Figure 13: The frequency of all main diagnoses in the arthropathy-dominant musculoskeletal category of autoimmune diseases is similarly affected by the exact length of the pathological CAG triplet expansion in people with the HD mutation** (A) Pie chart showing the distribution of the different diagnoses of the group of participants with arthropathy-dominant autoimmune diseases of musculoskeletal and connective tissues (AID_arthro_), specific diagnoses as given as shades of ultramarine while unspecific codes are given in shades of light blue (RA = rheumatoid arthritis, n.e.c = not elsewhere classified). In case of intra-category double-diagnoses, the more diagnosis was kept, if both were either specific or unspecific the more frequent AID_arthro_. The percentages are given within and the total number below the graph ~~are given~~. (B) Relative percentage of common specific and unspecific as well as rare AID_arthro_ diagnoses in control participants (CP) compared to people with the Huntington mutation (PwHD, Arthr. unspec. = Arthritis unspecified, AS = Ankylosing spondylitis) color-coded as in A, rare unspecific and specific AID_arthro_ were combined to “Other”. (C) Percentages of PwHD with the most frequent unspecific AID_arthro_ diagnosis (Arthritis, unspecified), the two most frequent specific AID_arthro_ diagnoses (RA and AS) and the other less frequent specific and unspecific AID_arthro_ combined (other arthropath. musc.) plotted separately grouped according to the length of the CAG triplet expansion (CAG) as indicated. (D) Similar data as in B but the participants with RA and SA where combined to a group with frequent specific AID_arthro_ diagnoses (RA/SA), while the patients with other AID_arthro_ and the diagnosis “arthritis, unspecified” were combined to the group “other/unsp.arthr.”. (B-D) The number of participants is given above within the bar graphs, the relative percentage when compared to the low pathological CAG repeat group (36-40) is given above in C and D. Statistical analysis was performed using Fisher’s exact tests with the p-values not adjusted for the number of diagnoses groups in B and C; n.s.= not significant, *p<0.05, **p<0.01, ***p<0.0001. Borderline p-values are given as exact values,


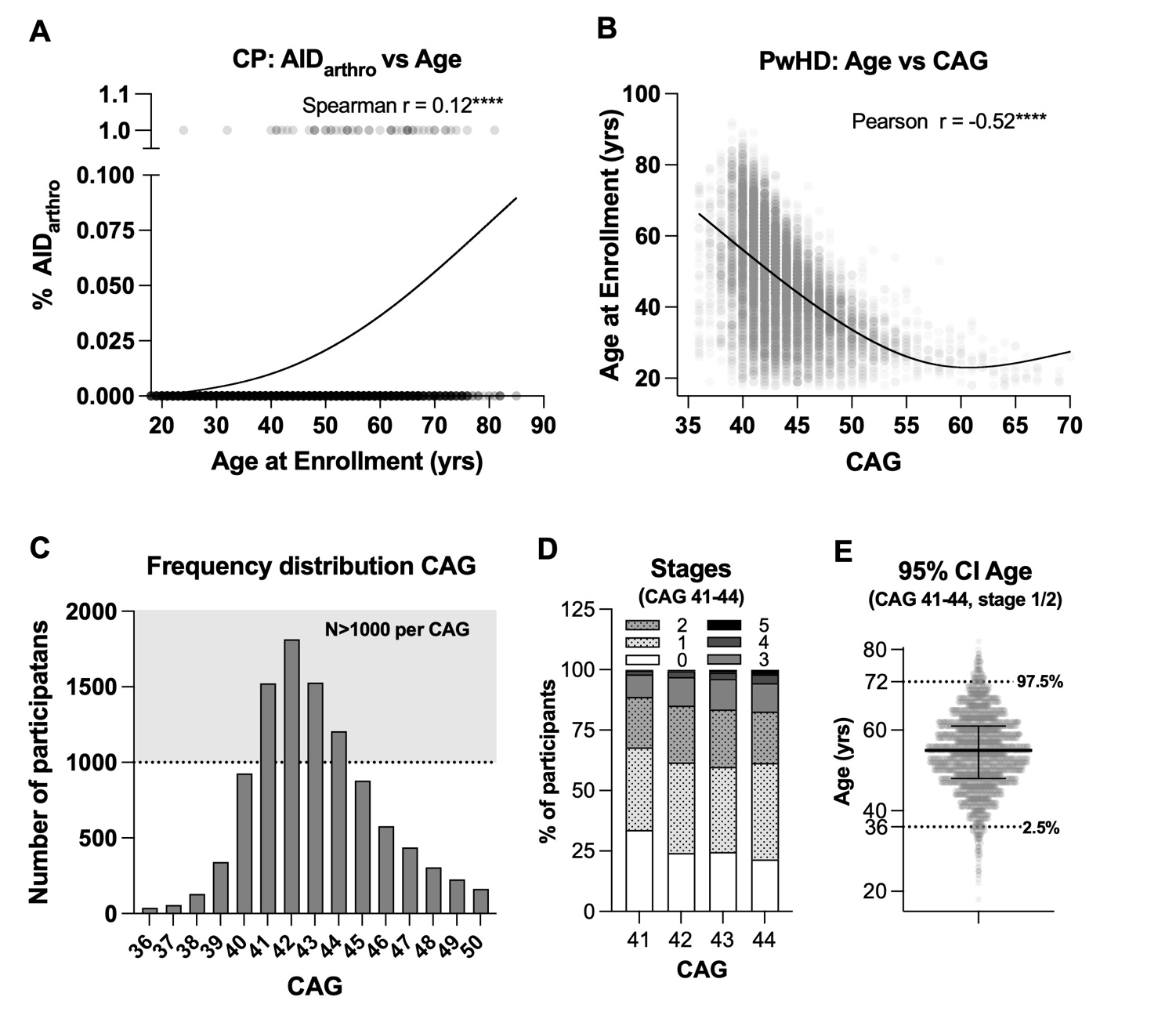


**Supplementary Figure 14: Rational for the core cohort of people with the Huntington mutation to assess the CAG dependency of the AID_arthro_ frequency. (**A) The frequency of arthropathy-dominant autoimmune diseases of musculoskeletal and connective tissues (AID_arthro_) is age-dependent in control participants (CP). Presence (1) or absence (0) of an AID_arthro_ diagnosis plotted against age at enrollment in 2,477 CPs. (B) The age at enrollment is plotted against the individual CAG repeat length in the PwHD cohort. (A/B) Black line represents results of a spline regression analysis. (C) Frequency distribution of specific CAG-repeat lengths in the PwHD cohort. CAG repeat numbers less than 36 and higher than 50 are omitted for clarity. The shaded areas indicate CAG group sizes of >1000. (D) Frequency distribution of presymptomatic (0) and manifest PwHD with the different functional stages of HD (1-5). Stages 1 and 2 are dotted. (E) Distribution of the age at enrollment in the subcohort of PwHD with 41-44 CAG repeats and stages 1 or 2. The median and interquartile range of the subcohort is indicated as are the 2.5^th^ (36 years) and 95^th^ percentiles (72 years). Statistical analysis in (A) was performed by Spearman rank and in (B) by Pearson correlation. The r is given within the graphs; ****p<0.0001.


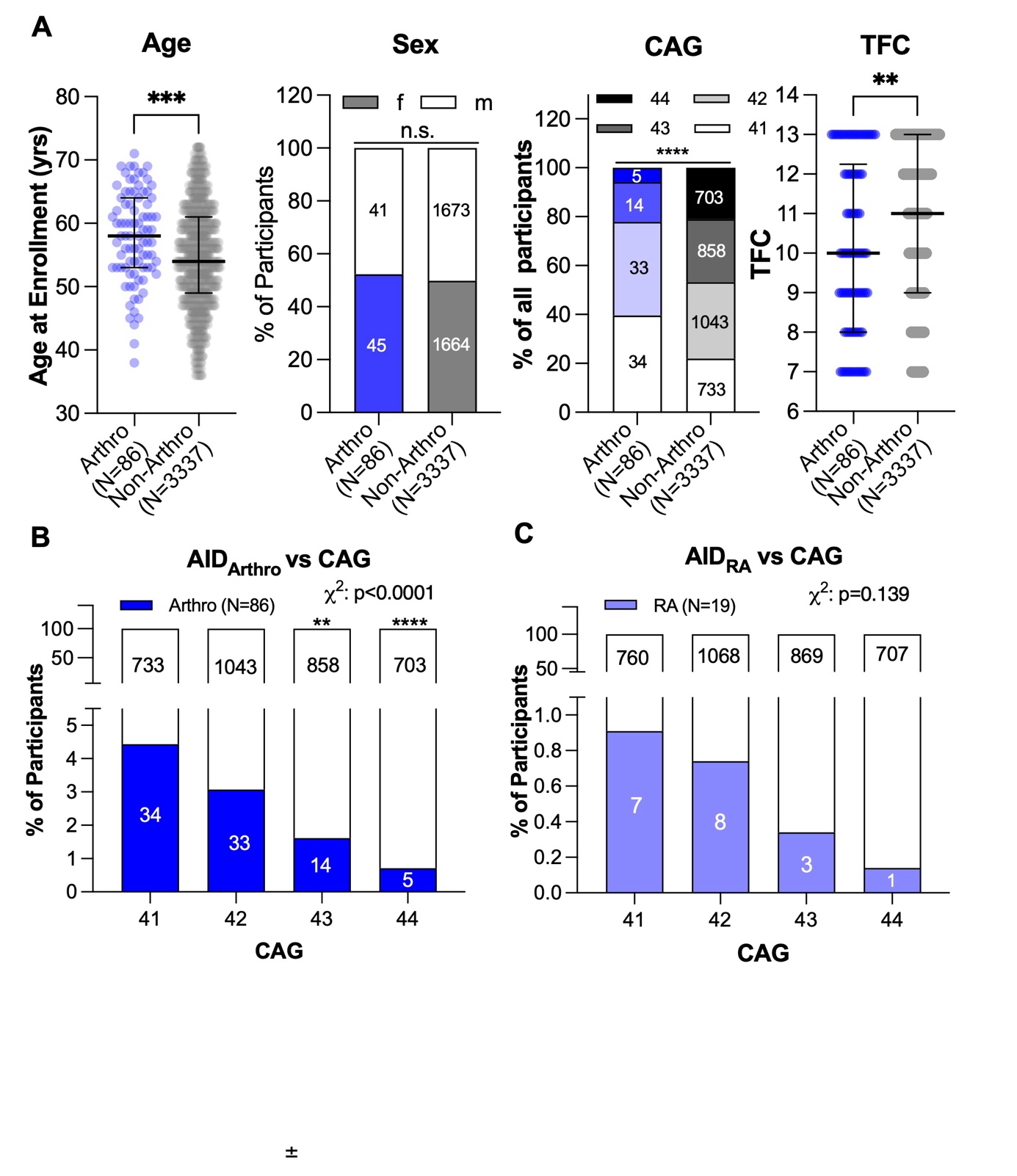


**Supplementary Figure 15: Characteristics of the core cohort of people with the HD mutation with 41-44 CAG repeats, stages 1/2 and age at enrollment of 36 to 72 yrs.** (A) Age (left), sex (f=female, m=male, middle left), CAG number (middle right) and total functional capacity score (TFC, right) distributions in people with the HD mutation with an arthropathy-dominant autoimmune diseases of musculoskeletal and connective tissues (AID_Arthro_, Arthro) or not (Non-Arthr.). The group sizes are indicated. (B/C) Frequencies of participants with either AID_arthro_ (B) or with the specific diagnosis of rheumatoid arthritis (RA, C) at different CAG repeats numbers. Statistical analysis was performed by Whitney-Mann U tests in the left and right graphs of A and Fisher’s exact tests in A, middle panels left and right, B and C. In B/C the p-values for a global test are indicated (x^2^) as are the Bonferroni-corrected p-values for pairwise Fisher’s exact tests compared to the group with 41 CAG repeats when statistically significant; n.s.= not significant, *p<0.05, **p<0.001, ***p<0.0001, ****p<0.00001.


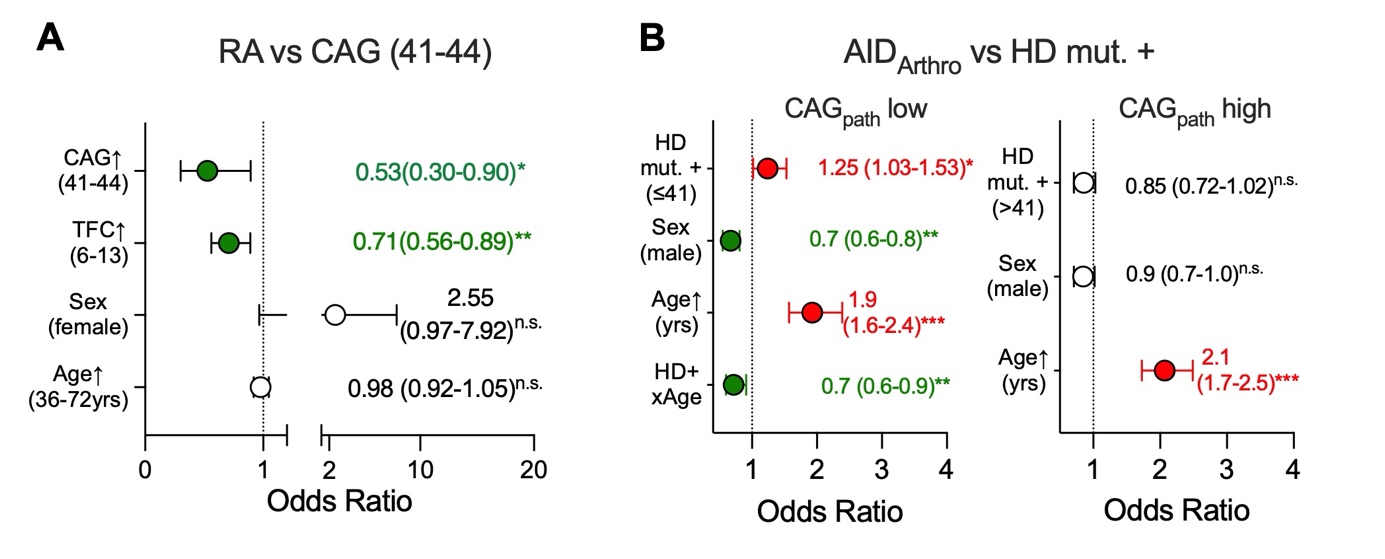


**Supplementary Figure 16: Higher CAG repeats reduce the risk for rheumatoid arthritis and arthropathic musculoskeletal autoimmune disease occur more frequently in people with the Huntington disease mutation with low pathological CAG repeats** (A) Forest plot of the odds ratios obtained by multiple logistic regression analysis (MLRA) of the influencing variables “higher CAG” (CAG↑), “higher total functional capacity” (TFC↑) score, “female sex” and “higher age” (Age↑) for the diagnosis of rheumatoid arthritis (RA) in the HD-mutation carrier core group with 41-44 CAG repeats, stages 1 or 2 and age at enrollment of 36 to72 years as described in Supplementary Figure 15. (B) Forest plots showing the odds ratios obtained by MLRA of the effect- and z-standardized influencing variables “HD mutation carriership” (HD mut. +), “male sex”, “higher age in years per SD” [age↑(yrs), ≤41 CAG: SD = 12.7 yrs, >41 CAG: SD = 15.3 yrs], and the interaction of HD carrier status and age (HD+ x age) for the diagnosis of an arthropathy-dominant AID of musculoskeletal and connective tissues (AID_arthro_) among all CPs and PwHDs with equal or less 41 CAG triplet repeats (left panel, CAG_path_low) or all CPs and those PwHD with >41 CAG repeats (right panel, CAG_path_high). The symbols and error bars indicate the mean and 95% CI of the odds ratios. (A/B) Variables associated with a decreased risk are green, red indicated an increased risk, those without a statistically significant effect are black and white. MLRA war performed without z-standardization and identification of interactions in A. In B MLRA war performed with dichotomous variables being effect-coded as -1 and 1 and continuous variables being z-standardized to enable the identification of interaction. The odds ratios are given as mean and 95% confidence interval; n.s.: not significant, *p<0.05, **p<0.001, ***p<0.001.
